# Photo‐Induced Synthesis of Ytterbium and Manganese‐Doped CsPbCl_3_ Nanocrystals for Visible to Near‐Infrared Photoluminescence with Negative Thermal Quenching

**DOI:** 10.1002/advs.202408927

**Published:** 2024-12-04

**Authors:** Xiaochen Fang, Zhuo Chen, Mandy Hei Man Leung, Biao Zheng, Liwei Wang, Meng An, Yusuke Asakura, Yusuke Yamauchi, Zhanhui Yuan

**Affiliations:** ^1^ College of Materials Engineering Fujian Agriculture and Forestry University Fuzhou 350002 China; ^2^ College of Mechanical and Electrical Engineering Shaanxi University of Science and Technology Xi'an 710021 China; ^3^ Department of Materials Process Engineering Graduate School of Engineering Nagoya University Nagoya Aichi 464–8603 Japan; ^4^ Fujian Key Laboratory of Functional Marine Sensing Materials College of Material and Chemical Engineering Minjiang University Fuzhou 350108 China; ^5^ Australian Institute for Bioengineering and Nanotechnology (AIBN) The University of Queensland Brisbane QLD 4072 Australia

**Keywords:** CsPbCl_3_ nanocrystals, Mn^2+^ and Yb^3+^ dopants, Negative thermal quenching, Photoinduction

## Abstract

Rare‐earth‐doped all‐inorganic perovskite applications for near‐infrared (NIR) emission are crucial for the construction of the next generation of intelligent lighting sources. However, the preparation of rare‐earth‐doped all‐inorganic perovskite is complex, and difficult to control, and the issue of thermal quenching poses significant challenges to its practical application. Here, in order to address these issues, a convenient photo‐induced synthesis method for CsPbCl_3_:Mn/Yb nanocrystals (NCs) is proposed by decomposing carbon tetrachloride with 365 nm light to provide chloride ions and regulate the formation of perovskite at room temperature. The negative thermal quenching in the NIR emission is achieved through the energy transfer between Mn and Yb. The emission intensity of Yb enhances 3.2 times when the temperature rises to ≈427 K. Furthermore, with the help of the orange emission from the Mn^2+^ ions and the NIR emission from the Yb^3+^ ions, visible to NIR light emitting diode (LED) devices are constructed and applied in orange light illumination and night vision imaging. This study enriches the preparation methods and chemical research on perovskite doping, which may open up new opportunities for the widespread application of perovskite‐based materials or device engineering.

## Graphical abstract

This paper presents a photo‐induced synthesis method for CsPbCl₃:Mn/Yb nanocrystals, achieved by decomposing carbon tetrachloride with 365 nm light at room temperature. The nanocrystals exhibit negative thermal quenching in near‐infrared emission through Mn‐Yb energy transfer and are applied in visible to near‐infrared LEDs for orange light illumination and night vision imaging.

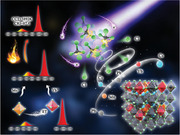

## 1 Introduction

Near‐infrared (NIR) light emitting diode (LED) devices are increasingly recognized for their broad applications, including night‐time navigation, secure optical communication systems, advanced medical imaging, non‐invasive diagnostics, and the assessment of food safety and quality.^[^ ^1^, ^2^, ^3^, ^4^ ^]^ The development of phosphor‐converted (pc) NIR LED device typically involves a NIR emitting phosphors and a standard 365 nm commercial UV LED chip. This integration offers several advantages, such as a compact form factor, cost‐effectiveness, and high energy efficiency.^[^ ^5^, ^6^ ^]^ The efficacy of NIR‐LED systems is critically dependent on the properties of the NIR luminescent materials employed.^[^ ^7^, ^8^, ^9^, ^10^ ^]^ However, the operation of a 365 nm commercial UV LED chip would generate plenty of heat, known as Joule heat, resulting in the decrease of luminescence in the phosphors from thermal quenching effect.^[^ ^11^, ^12^, ^13^ ^]^ For instance, the luminescence of Sr_3_Sc_4_O_9_:Cr^3+^ phosphors experiences a notable decrease at high temperature, which only retaining 70.4% of the initial intensity at 100 °C.^[^ ^14^ ^]^ The NIR emission of Cr^3+^ in YGa_3_(BO_3_)_4_:Cr^3+^ is only 62% of its original at 150 °C. These luminescent powder materials are significantly affected by high temperature, which limits their application in practical use.^[^ ^15^ ^]^ In response to these challenges, ongoing research studies are dedicated to developing advanced phosphor materials with improved thermal stability and higher photon conversion efficiency, including the exploration of new phosphor compositions, the optimization of the device's thermal management systems, and the design of innovative packaging solutions to reduce heat accumulation.

Incorporating lanthanide ions into all‐inorganic cesium lead halide perovskite nanocrystals (CsPbX_3_, X = Cl, Br, I) is undoubtedly the best choice to achieve NIR luminescence. As is well known, CsPbX_3_ nanocrystals (NCs) show broad chemical tunability, excellent charge transport properties, and high photoluminescence quantum yields (PLQYs), enabling promising applications in the field of optoelectronics, LEDs, and lasers.^[^ ^16^, ^17^ ^]^ However, common synthesis techniques for CsPbX_3_ NCs, such as hot injection method and supersaturated recrystallization method, often involves rigorous processes.^[^ ^18^, ^19^ ^]^ Specially, the hot injection method is prevalent to synthesize high‐quality CsPbX_3_ NCs, but it requires high temperature, vacuum environment, and inert atmosphere. The supersaturated recrystallization method offers a simpler synthesis process and could generate large quantities of products, but it produces a lot defects and leads to poor crystallization.^[^ ^20^ ^]^ On the other hand, the inherent instability of CsPbX_3_ NCs usually results in weak luminescence at high temperatures. To solve this problem, doping with metallic ions has been explored as an effective method to improve the optical properties of CsPbX_3_ NCs.^[^ ^11^, ^21^, ^22^, ^23^, ^24^ ^]^ Doping with Mn^2+^ ions gives emission in visible region with PLQY of 80%, while NIR emissions with PLQY higher than 100% as a result from the quantum cutting effect when doped with Yb^3+^ ions upon excitation by UV light. Furthermore, the Yb^3+^ activator can be triggered by energy transfer from the exciton of CsPbX_3_ NCs. Therefore, Mn^2+^/Yb^3+^ co‐doped CsPbCl_3_ NCs are a promising candidate for NIR LED devices, which could emit at visible to NIR range with high PLQY and exhibit strong resistance to thermal quenching.

Herein, a simple and facile photo‐induced synthesis method is proposed to prepare CsPbCl_3_:Mn/Yb NCs at room temperature, which simplifies the synthetic process, making it more efficient and user‐friendly but also allows for precise control over the formation of the products. The negative thermal quenching phenomenon in CsPbCl_3_:Mn/Yb NCs is realized, and the corresponding enhancement mechanism is revealed. Furthermore, the remarkable triple‐wavelength emissions of exciton recombination (ultraviolet/blue), Mn^2+^ (visible), and Yb^3+^ (NIR) in CsPbCl_3_:Mn/Yb NCs offer a versatile candidate phosphor for Vis‐NIR LED device. As a demonstration, a pc‐Vis‐NIR LED device is designed using CsPbCl_3_:Mn/Yb NCs, which shows promising applications in biomedical imaging, nondestructive testing, and night‐vision devices.

## 2 Results and Discussion

**Figure 1:**
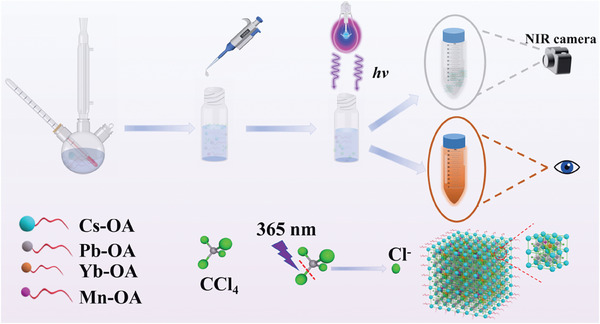
Schematic diagram of the synthesis of CsPbCl_3_:Mn/Yb NCs via a photo‐induced strategy. Cs‐OA, Pb‐OA, Yb‐OA, and Mn‐OA precursors were synthesized. Controlled release of Cl^−^ ions was initiated with irradiation at 365 nm and the released Cl^−^ ions from CCl_4_ was interacted with Cs^+^, Pb^2+^, Yb^3+^, and Mn^2+^ ions, leading to the spontaneous nucleation and growth of CsPbCl_3_:Mn/Yb NCs at room temperature.

Our synthesis strategy for CsPbCl_3_:Mn/Yb NCs involves a photo‐induced approach illustrated in **Figure** **1**. Cesium carbonate (Cs_2_CO_3_), manganese acetate (Mn(CH_3_COO)_2_·4H_2_O), lead acetate (Pb(CH_3_COO)_2_·3H_2_O), and ytterbium acetate (Yb(CH_3_COO)_2_·4H_2_O) were each heated under vacuum in the presence of oleic acid and oleylamine to form Cs‐OA, Pb‐OA, Yb‐OA, and Mn‐OA, respectively, for subsequent use. Then, cesium oleate, lead oleate, manganese oleate, and ytterbium oleate were dissolved in carbon tetrachloride (CCl_4_), utilizing CCl_4_ as the solvent medium and halide precursor. Under the irradiation of 365 nm UV light, the C─Cl bond of CCl_4_ undergoes dissociation, progressively releasing Cl^−^ in a controlled manner.^[^ ^25^ ^]^ The released Cl^−^ ions then engage in a dynamic interplay with Cs^+^, Pb^2+^, Yb^3+^, and Mn^2+^ ions in the solvent, leading to the spontaneous nucleation and growth of CsPbCl_3_:Mn/Yb NCs at room temperature. Under 365 nm light, the colorless and nonluminous CCl_4_ solution exhibited orange photoluminescence (PL). In contrast, in the absence of light or under non‐photoexciting ambient conditions, the haloalkane solutions maintain transparency and lack luminescence, unveiling the pivotal role of photoinduction in the synthesis process CsPbCl_3_:Mn/Yb NCs.

**Figure 2:**
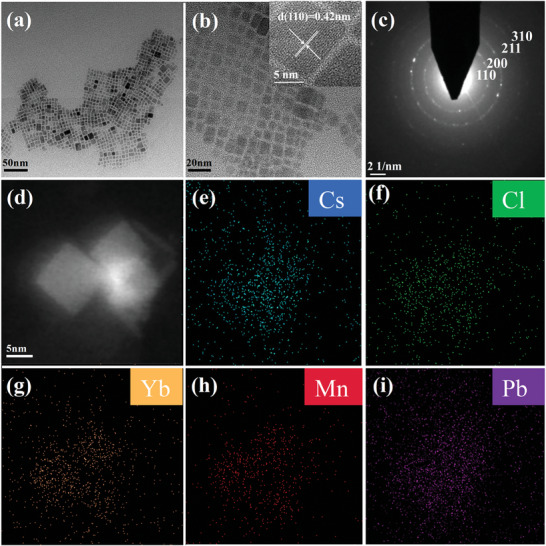
a,b) TEM image and inset in (b) is the HRTEM micrograph of an individual CsPbCl_3_:Mn/Yb particle. c) SAED pattern, d) HAADF‐STEM image, and e–i) EDX mappings in typical CsPbCl_3_:Mn/Yb NCs.

Morphological and crystallographic characterization of the Mn/Yb co‐doped CsPbCl_3_ NCs, synthesized via the photo‐induced method, is depicted in **Figure** **2**. Transmission electron microscopy (TEM) images reveal that the CsPbCl_3_:Mn/Yb NCs have a pure cubic structure with a uniform size distribution of ≈6 nm (Figure 2a,b). High‐resolution TEM (HRTEM) images confirm the single‐crystalline nature of the NCs, evidenced by distinct lattice fringes. A characteristic d‐spacing of 0.42 nm (inset of Figure 2b), corresponding to the (110) planes of the cubic perovskite structure, is also observed. Selected area electron diffraction (SAED) patterns further confirm the pure cubic crystalline structure of the NCs (Figure 2c). The High‐Angle Annular Dark Field Scanning TEM (HAADF‐STEM) images (Figure 2d) and energy‐dispersive X‐ray (EDX) elemental mapping (Figure 2e–i) of CsPbCl_3_ NCs show a uniform distribution of Cs, Pb, Cl, Mn, and Yb signals, verifying the successful incorporation of Mn^2+^ and Yb^3+^ dopants into the CsPbCl_3_ lattice. The electron paramagnetic resonance spectrum of CsPbCl_3_:Mn/Yb NCs is shown in Figure S1 (Supporting Information). The Mn^2+^/Yb^3+^ co‐doped CsPbCl_3_ NCs exhibit a set of six hyperfine splitting peaks characteristic of nuclear electron transitions, with an average splitting constant of 86.6 G and a g‐factor value of 2.0. This indicates that the Mn^2+^ ions have been successfully incorporated into the octahedral coordination environment of the cubic perovskite lattice.

**Figure 3:**
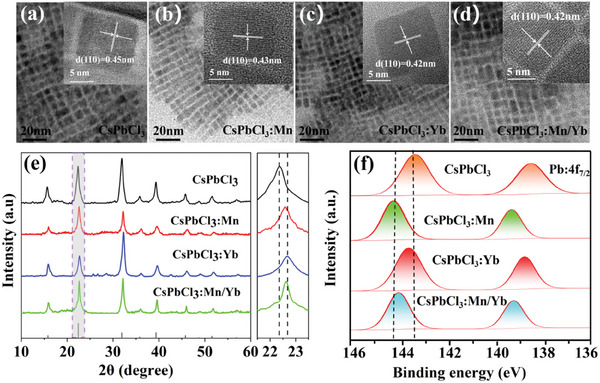
TEM images of a) CsPbCl_3_, b) CsPbCl_3_:Mn, c) CsPbCl_3_:Yb, and d) CsPbCl_3_:Mn/Yb NCs. Insets are the HRTEM micrographs of an individual particle. e) XRD patterns and f) XPS spectra for Pb:4f_7/2_ in CsPbCl_3_, CsPbCl_3_:Mn, CsPbCl_3_:Yb, and CsPbCl_3_:Mn/Yb NCs.

To investigate the impact of Mn and Yb doping and co‐doping on the structural characteristics of CsPbCl_3_ NCs, the samples of CsPbCl_3_, CsPbCl_3_:Mn, CsPbCl_3_:Yb, and CsPbCl_3_:Mn/Yb NCs were synthesized by the photo‐induced method described earlier. TEM images reveal that all samples exhibit a similar cubic morphology (**Figure** **3**a–d), with average edge lengths of 12.19 ± 2.54 nm for CsPbCl_3_, 7.74 ± 2.28 nm for CsPbCl_3_:Mn, 5.67 ± 2.18 nm for CsPbCl_3_:Yb, and 6.16 ± 2.08 nm for CsPbCl_3_:Mn/Yb, as depicted in Figure S2 (Supporting Information). The HRTEM images indicate that all samples are single crystalline with well‐resolved lattice fringe, and the d‐spacings decrease with the doping of Mn and Yb (insets of Figure 3a–d). This is due to the smaller ionic radii of Mn and Yb compared to Pb, and their incorporation by substituting Pb^2+^ ions in CsPbCl_3_, which leads to a contraction of the CsPbCl_3_ lattice and a reduction in size. The EDX spectrum can clearly reveal the presence of elements such as Cs, Pb, and Cl, indicating the successful synthesis of CsPbCl_3_NCs. Notably, in CsPbCl_3_:Mn and CsPbCl_3_:Mn/Yb, peaks corresponding to Mn are detected, and similarly, peaks for Yb are observed in both CsPbCl_3_:Yb and CsPbCl_3_:Mn/Yb. This demonstrates the successful doping of Mn or Yb into CsPbCl_3_ (Figure S3, Supporting Information). The X‐ray diffraction (XRD) patterns confirm that all synthesized products align with the cubic phase of CsPbCl_3_ (JCPDS No. 75‐0408) (Figure 3e), indicating that the introduction of Mn^2+^ and Yb^3+^ dopants does not cause any impurity phases.^[^ ^26^, ^27^ ^]^ Notably, the subtle shift of the Bragg diffraction peak toward higher angles suggests that the larger Pb^2+^ ions (1.19 Å, CN = 6),^[^ ^28^, ^29^ ^]^ have been effectively replaced by the smaller Mn^2+^ (0.97 Å, CN = 6)^[^ ^30^, ^31^ ^]^ and Yb^3+^ (1.02 Å, CN = 6) ions.^[^ ^32^ ^]^ This substitution leads to a decrease in the lattice constant from 5.631 to 5.557 Å, further implying the lattice shrinkage caused by Mn^2+^ and Yb^3+^ doping (Table S1, Supporting Information). However, the XRD shift of CsPbCl_3_:Yb is significantly greater than that of CsPbCl_3_:Mn. We attribute this to the fact that under conditions of insufficient chlorine, the incorporation of Mn is less than the incorporation of Yb.^[^ ^33^ ^]^ Furthermore, XPS analyses were conducted on the CsPbCl_3_, CsPbCl_3:_Mn, CsPbCl_3_:Yb, and CsPbCl_3_:Mn/Yb NCs. The expected characteristic signal peaks corresponding to Cs 3d, Pb 4f, and Cl 2p were identified. It is worth noting that the presence of Yb 4d peaks in CsPbCl_3_:Yb and CsPbCl_3_:Mn/Yb, as well as the Mn 2p peaks in CsPbCl_3_:Mn and CsPbCl_3_:Mn/Yb, can be distinctly observed, further confirming the incorporation of Mn and Yb (Figure S4, Supporting Information). As depicted in Figure 3f, the shift of the Pb 4f peak toward higher binding energies upon Mn/Yb doping indicates a change in the local electronic environment surrounding the Pb^2+^ ions. The shift of the Pb 4f peak toward higher binding energies after Mn/Yb doping is attributed to changes of the local electronic environment surrounding the Pb^2+^ ions, where the larger ionic radii of Pb^2+^ ions are partially substituted by the smaller ionic radii of Mn^2+^ and Yb^3+^ ions.

**Figure 4:**
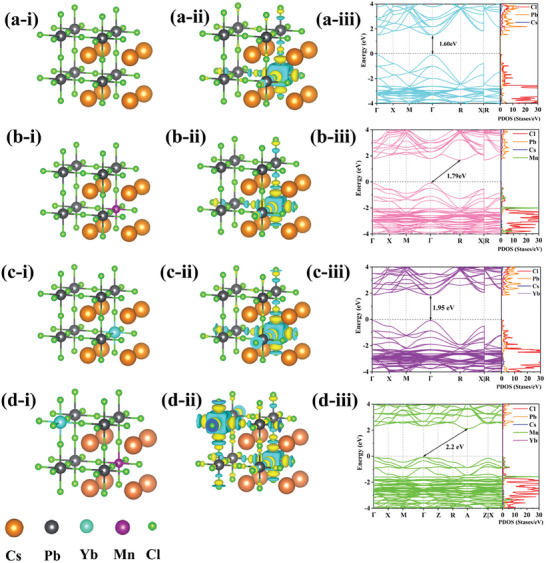
The theoretical calculation of a) CsPbCl_3_, b) CsPbCl_3_:Mn, c) CsPbCl_3_:Yb, and d) CsPbCl_3_:Mn/Yb NCs. (i) crystal structure (ii) calculated deformation charge density (iii) the band structure and partial density of states (PDOS).

To further understand the origin of the changes in the electronic structure of CsPbCl_3_ NCs induced by Mn^2+^ and Yb^3+^ doping, we have investigated the lattice structure, charge density difference, band structure, and projected density of states of undoped, Mn^2+^and Yb^3+^ doped CsPbCl_3_ NCs using density functional theory. The first‐principles calculation details can be found in Supplementary Materials. Based on recent work,^[^ ^34^ ^]^ Mn^2+^ and Yb^3+^ have replaced Pb^2+^ in CsPbCl_3_, adopting an octahedral Cl^−^ coordination environment in **Figure** **4**a‐i–d‐i, which is attributed to  their formation of lower energy states, high stability, and similar valence states and ionic radii to that of Pb^2+^. We have discussed the impact of Mn and Yb at different positions in the CsPbCl_3_ lattice, with the constructed lattice structures shown in Figure S5a–c (Supporting Information). The formation energy of Figure S5a,b (Supporting Information) are ‒154.77 eV, while that of Figure S5c (Supporting Information) is ‒154.88 eV, indicating that the lattice structure of Figure S5c (Supporting Information) is the most stable, and the doping of Mn and Yb is most likely to exist in a diagonal body‐to‐body form. Furthermore, we calculated the deformation charge density and band structure of different samples and found that the lattice structure of Figure S5c (Supporting Information) also has stronger charge migration capabilities and a larger bandgap (Figure S5d–i, Supporting Information). Figure 4a‐ii–d‐ii show the charge density differences of CsPbCl_3_, CsPbCl_3_:Mn, CsPbCl_3_:Yb, and CsPbCl_3_:Mn/Yb NCs, which were extracted from the charge density differences between the self‐consistent pseudo charge density and the superposition of atomic charge densities. The electron gain and loss are denoted by yellow and cyan colors, respectively. Obviously, it was observed that the Cl tends to gain electrons while Pb, Mn, and Yb tend to lose electrons. Yb is particularly prone to losing electrons compared to Mn and Pb. In Figure 4a‐iii–d‐iii, the states around the band gap of undoped CsPbCl_3_ NCs are primarily derived from the Pb 6*p* and Cl 3*p* orbitals, with a negligible contribution of the Cs orbitals. With the incorporation of Mn and Yb, the bandgap of the CsPbCl_3_ NCs host gradually increases, which can be attributed to the enhanced Pb‐Cl interactions due to lattice contraction. Mn predominantly contributes to the valence band, whereas Yb does not significantly participate in the band structure. Notably, the incorporation of Mn results in a change from a direct bandgap to an indirect bandgap, as Mn doping induces a *d*‐band to *d*‐band transition.^[^ ^35^ ^]^ As reported in previous studies, the bond lengths for Yb‐Cl and Mn‐Cl are 2.58 and 2.47 Å, respectively, which is smaller than the Pb‐Cl bond distance of 2.83 Å.^[^ ^36^, ^37^ ^]^ The changes in the bandgap align with the XRD results on the structural properties shown in Figure 3e, confirming that Mn^2^⁺ and Yb^3^⁺ ions have been successfully doped into the CsPbCl_3_ lattice, with substitution occurring exclusively at the Pb^2^⁺ sites.

**Figure 5:**
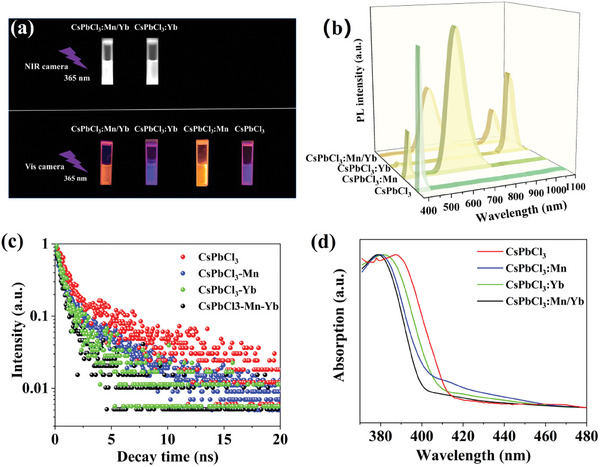
a) The luminescent photographs of solutions containing CsPbCl_3_, CsPbCl_3_:Mn, CsPbCl_3_:Yb, and CsPbCl_3_:Mn/Yb NCs under UV light irradiation. b) PL spectra, c) PL decay curves, and d) UV–vis spectra of CsPbCl_3_, CsPbCl_3_:Mn, CsPbCl_3_:Yb, CsPbCl_3_:Mn/Yb NCs.

**Figure** **5**a shows photographs of CsPbCl_3_, CsPbCl_3_:Mn, CsPbCl_3_:Yb, and CsPbCl_3_:Mn/Yb NCs captured by a visible camera and a NIR camera. The NCs doped with Mn (CsPbCl_3_:Mn and CsPbCl_3_:Mn/Yb) NCs emit orange luminescence, while the CsPbCl_3_:Yb and CsPbCl_3_ NCs show violet luminescence. The PL spectrum of the pristine CsPbCl_3_ NCs reveals a narrow violet band edge exciton recombination emission at ≈400 nm.^[^ ^38^ ^]^ The incorporation of Mn^2+^ and Yb^3+^ dopants into the CsPbCl_3_ lattice introduces two additional broadband emissions at orange (≈601 nm) and NIR (≈980 nm) wavelengths (Figure 5b), corresponding to the d‐d transitions of Mn^2+^ (^4^T_1g_→^6^A_1g_ ) and the f‐f transitions of Yb^3+^ (^2^F_5/2_→^2^F_7/2_), respectively.^[^ ^39^ ^]^ Notably, the intensity and lifetime of exciton recombination decrease significantly after introducing Mn^2+^ and Yb^3+^ dopants, indicating the presence of an additional energy transfer (ET) from the exciton to Mn^2+^ and Yb^3+^ dopants (Figure 5c and Table S2, (Supporting Information)). Figure 5d illustrates the blue shift of the exciton absorption edge in the presence of Mn^2+^ and Yb^3+^ dopant ions, which may be attributed to that the incorporation of Mn^2+^ and Yb^3+^ dopants results in the contraction of the host lattice of CsPbCl_3_. Figure S6a–d (Supporting Information) presents the PL spectral tests conducted for the PLQY, and calculated the PLQY of each sample using formula^[^ ^40^ ^]^:

(1) $$\mathrm{PLQY}=\left(\int \;L_{\mathrm{direct}}-\int \;L_{\mathrm{blank}}\right)/\left(\int \;E_{\mathrm{without}}-\int \;E_{\mathrm{direct}}\right)$$

where L_direct_ is the complete emission spectrum of the sample collected by using the integrating sphere, L_blank_ is the emission spectrum of the blank sample, E_direct_ is the emission spectrum of the excitation light, recorded with the sample in place, and E_without_ is the emission spectrum of excitation light, recorded with the equipment blank in place. The calculated PLQY is shown in Table S3, (Supporting Information). Due to the ET from excitons to Mn and Yb, the PLQY of excitons significantly decreases from 6.52% to 0.58% upon doping with both Mn and Yb when compared with the CsPbCl_3_ host NCs. Furthermore, the PLQY of Yb is 100.48%. The PLQY of Yb in the CsPbCl_3_:Mn/Yb sample exceeding 100% indicates that the ET from excitons to Yb^3^⁺dopants is a typical quantum‐cutting process, similar to the previously reported cases of CsPbCl_3_:Yb prepared by other methods.^[^ ^24^, ^41^ ^]^ To confirm this, photoexcitation power‐dependent NIR PL spectra of CsPbCl_3_:Yb were recorded (Figure S7a, Supporting Information). Theoretically, NIR emission intensity, *I*_Yb_, depends on the photoexcitation power, *P* ^n^, according to the relationship of *I*_Yb_∞*P* ^n^, where *n* is equal to 0.5 for the nonlinear quantum cutting process.^[^ ^11^ ^]^ As shown in Figure S7b (Supporting Information), the slope of the double logarithmic plot of NIR integrated intensity versus pump laser power is fitted to be 0.64. This sub‐linear fitting confirms the dominant quantum cutting luminescence process in the CsPbCl_3_:Yb NCs, and the deviation from the theoretical value of 0.5 is probably attributed to the saturation population of Yb^3+ 2^F_5/2_ excited state stemming from its long lifetime.^[^ ^42^ ^]^

**Figure 6:**
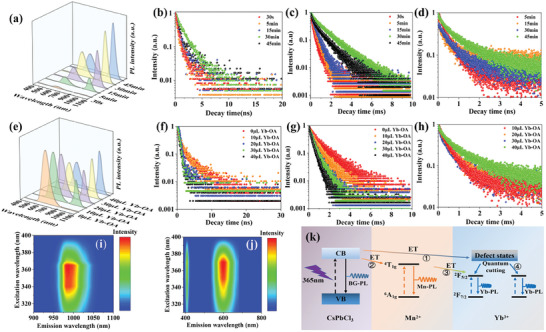
a) PL spectra and b–d) PL lifetime decay curves of the exciton recombination, Mn, Yb in CsPbCl_3_:Mn/Yb NCs under different UV light irradiation times. e) PL spectra and f–h) PL lifetime decay curves of the exciton recombination, Mn emission, Yb emission in CsPbCl_3_:Mn/Yb NCs doped with various Yb^3+^ contents. Pseudocolor maps of i) Yb emission and j) exciton recombination and Mn emission dependence on the excitation and emission wavelengths. k) Schematic mechanisms of the energy transfer process within CsPbCl_3_: Mn/Yb NCs.

We further characterized the optical properties of CsPbCl_3_:Mn/Yb NCs prepared by the photoinduction method under different conditions, including the irradiation time of UV light, the quantities of Mn‐OA, Yb‐OA, and CCl_4_. **Figure** **6**a shows the PL emission intensities of exciton recombination, Mn and Yb in CsPbCl_3_:Mn/Yb NCs under different irradiation times of UV light. Under the condition of UV light irradiation for 30 s, the luminescence of both excitons and Mn was detected but not Yb. This is attributed to the fact that Mn is more likely to capture the energy of excitons than Yb. When the reaction time increases to 30 min, the emission intensities from exciton recombination and Mn^2+^ emission reach their maximum. As shown in Figure S8 (Supporting Information), the Bragg diffraction peaks shift toward higher angles and become sharper from 30 s to 30 min of light exposure. The enhanced optical properties of CsPbCl_3_:Mn/Yb with increasing illumination time are attributed to the improvement in crystallinity and the gradual incorporation of Mn and Yb. The calculated lattice constant decreases from 5.674 to 5.515 Å with increased light exposure from 30 s to 30 min (Table S4, Supporting Information). When the reaction time surpasses 30 min, the emission of Mn and excitons begins to decrease, while the emission of Yb continues to increase, which may be due to two ET processes: 1) from exciton to Yb^3+^ ion and 2) from Mn^2+^ ion to Yb^3+^ ion. Further research was conducted on the lifetimes of excitons, Mn, and Yb at different reaction times. Due to the reduction of defects and the gradual formation of CsPbCl_3_:Mn/Yb NCs, the PL lifetimes of excitons, Mn, and Yb gradually increased with the increase in reaction time within 30 min. There is an ET relationship between excitons and Mn with Yb, therefore, when the reaction time exceeds 30 min, the PL lifetime of excitons and Mn decreases. (Figure 6b–d and Tables S5–S7, Supporting Information).

Furthermore, the impact of CCl_4_ on the optical properties of CsPbCl_3_:Mn/Yb NCs is also investigated (Figure S9 and Tables S8–S10, Supporting Information). The emission intensities of excitons, Mn, and Yb increase with the increasing amount of CCl_4_, indicating that CCl_4_ can effectively facilitate the formation of CsPbCl_3_:Mn/Yb NCs. With increased amount of CCl_4_, the PL lifetime of excitons gradually decreases due to the ET from excitons to Mn and Yb. The PL lifetime of Mn exhibits a declining trend due to the concentration quenching induced by the increased Mn^2+^–Mn^2+^ interactions. Figure S10 (Supporting Information) shows the XRD patterns of CsPbCl_3_:Mn/Yb NCs prepared with various amount of CCl_4_. It was found that the crystallization performance is best with 3 mL, and as the amount of CCl_4_ increases, the Bragg diffraction peaks shift toward higher angles. This is due to the increase in CCl_4_, which promotes the incorporation of Mn/Yb, leading to lattice contraction (Table S11, Supporting Information).

Furthermore, we investigated the impact on PL with incrementally increasing the amount of Yb‐OA while the amount of Mn‐OA was fixed at 10 µL (Figure 6e–h). The PL intensity of Yb^3^⁺ gradually increased, with its lifetime extending from 0.21 to 1.05 ms. In contrast, the PL intensity of Mn^2^⁺ steadily decreased, and its lifetime shortened from 1.00 to 0.36 ms. This is due to the ET between Mn^2+^ and Yb^3+^ ions (Figure 6e–h and Tables S12–S14, Supporting Information). As the concentration of Yb‐OA increases, the XRD pattern shifts toward higher angles. This shift is attributed to the gradual lattice contraction of the CsPbCl_3_:Mn/Yb NCs as the concentration of Yb^3+^ ions increases (Figure S11 and Table S15, Supporting Information). It is worth noting that under the condition of 40 µL Yb‐OA, the PLQY of Yb is 102.5%, and the total PLQY reaches 125.3%, which may be caused by the quantum cutting of Yb (Figure S12, Supporting Information). Conversely, in the CsPbCl_3_:Mn/Yb NCs synthesized with 10 µL of Yb‐OA and increasing Mn^2+^ content, the PL intensity of both Yb^3+^ and Mn^2+^ monotonically decreases (Figure S13a, Supporting Information). This is due to the higher likelihood of Mn capturing the energy transferred by excitons, thereby almost completely quenching the luminescence of Yb when there is an excess of Mn. However, the excessive concentration of Mn^2+^ leads to enhanced coupling interactions between Mn^2+^ ions, resulting in the quenching of its PL and a reduction in the PL lifetime of Mn^2+^ (Figure S13b–d and Tables S16–S18, Supporting Information).^[^ ^43^, ^44^ ^]^ The XRD patterns, as anticipated, shift toward higher angles. This is attributed to the lattice contraction that occurs as the doping level of Mn increases (Figure S14 and Table S19, Supporting Information).

Figure 6i,j shows the 3D excitation‐emission matrix PL spectra of CsPbCl_3_:Mn/Yb NCs. With varying excitation wavelengths, there is no significant change in the emission positions of Mn^2+^ and Yb^3+^, indicating a uniform distribution of dopants. Moreover, their excitation wavelengths all peak ≈365 nm, suggesting the existence of ET relationships between excitons and Yb as well as Mn.^[^ ^45^ ^]^ Figure 6k illustrates the complex processes of photoexcitation, ET, and radiation paths in Mn^2+^/Yb^3+^ co‐doped CsPbCl_3_ NCs. Under ultraviolet excitation, excitons, as electron‐hole pairs, are initially produced within the CsPbCl_3_ host. A portion of these excitons recombines at the band edge, leading to the observed bandgap. With the introduction of Mn^2+^ ions, a subset of the excited electrons undergoes resonant Dexter‐type energy transfer to the excited state (^4^T_1_ _g_) of the Mn^2+^ dopant.^[^ ^26^ ^]^ For the Yb^3+^ dopant, due to the isoelectronic nature of Yb^3+^ ions in the CsPbCl_3_ host, internal lattice defects become involved in the photoluminescence relaxation process. Specifically, the energy transfer initially occurs from the excitons of the host to localized defects, followed by transfer to adjacent Yb^3+^ ions, culminating in a quantum‐cutting process.^[^ ^46^ ^]^ Additionally, direct energy transfer from the ^4^T_1_ _g_ electronic state of Mn^2+^ ions to the ^2^F_5/2_ state of Yb^3+^ ions may also occur within the perovskite NCs.

In addition, we explored the impact of different storage times and different light exposure times on the optical properties of CsPbCl_3_:Mn/Yb. As shown in Figure S15a (Supporting Information), with exposure to UV light, the emissions of excitons, Mn, and Yb in CsPbCl_3_:Mn/Yb decrease accordingly. This is attributed to the fact that CsPbCl_3_:Mn/Yb NCs are prone to decomposition under UV light, leading to the destruction of the NCs structure and thus weakening its luminescent performance. After 24 h of UV light exposure, the emission of excitons, Mn, and Yb is maintained at 38.8%, 56.5%, and 60.3% of their original emission, repectively. Figure S15b (Supporting Information) demonstrates the influence of storage time at room temperature on the PL spectra of CsPbCl_3_:Mn/Yb NCs, of which the emissions of excitons, Mn, and Yb decrease with the increase of storage time. We attribute this to the fact that oxygen can damage the surfaces of NCs through chemical reactions, thereby removing their ligands and quenching their emissions. On the eighth day, the emissions of excitons, Mn, and Yb are 16.3%, 49.5%, and 80% of their original emissions, respectively.

To further investigate the effects of Mn doping and temperatures on the PL of CsPbCl_3_:Yb NCs, we compared the PL spectra and lifetime decay of CsPbCl_3_:Yb and CsPbCl_3_:Mn/Yb NCs at various temperatures. For CsPbCl_3_:Yb NCs, as the temperature increases from 77 to 297 K, the PL intensities of the excitons decrease with increasing temperature (Figure S16a, Supporting Information). The lifetime measurement results for probing the exciton recombination dynamics are shown in Figure S16b (Supporting Information), and the corresponding fitted decay lifetimes are listed in Table S20 (Supporting Information). As the temperature rises from 77 to 297 K, the exciton lifetime gradually increases, which is primarily due to the formation of free carriers with slower recombination rates produced by the fission of bound excitons.^[^ ^11^ ^]^ The dynamic behavior is also reflected in the biexponential characteristics of the lifetime curve, where the higher the temperature, the more pronounced the biexponential fitting becomes.^[^ ^47^ ^]^ The lifetime of Yb decreases with increasing temperature due to an increase in non‐radiative transitions (Figure S16c and Table S21, Supporting Information). Importantly, at lower temperatures, the Yb emission spectrum exhibits a distinct structure corresponding to the electronic transition between the crystal field‐split ^2^F_5/2_ and ^2^F_7/2_ states. Notably, the Stark splitting peaks observed in CsPbCl_3_:Yb NCs are very similar to those in single crystals doped with Yb^3+^.^[^ ^24^ ^]^ This indicates that the Yb^3+^ activator is primarily located within the CsPbCl_3_ lattice rather than on the surface of the NCs. For CsPbCl_3_:Mn/Yb NCs, in addition to observing similar exciton recombination dynamics and changes in Yb^3+^ PL, while there is an additional emission from Mn^2+^ (Figure S17 and Tables S22–S24, Supporting Information). The enhancement in the emission from Mn^2+^ as the temperature increases and a blue shift in the Mn^2+^ emission is observed, which can be explained by the competitive recombination from the near‐band‐edge and ^4^T_1g_ state to the ^6^A_1_ state following ET in the host CsPbCl_3_, as well as the reduction in crystal field strength caused by the thermal expansion of the host lattice and the thermal activation of the vibrational hot band.^[^ ^45^, ^48^ ^]^ The lifetime spectra of Mn^2+^ ions exhibit an approximately single exponential decay across various temperatures (Figure S17d and Table S23, Supporting Information), which may be associated with thermal quenching. This behavior suggests that the Mn^2+^ ions are situated within a uniform crystal field environment.^[^ ^49^ ^]^

**Figure 7:**
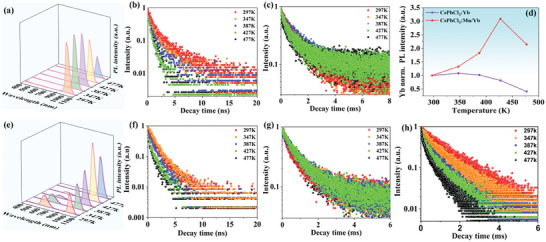
a) PL spectra and b,c) decay curves of excitons recombination and Yb emission in CsPbCl_3_:Yb NCs under different temperatures (297–477 K). d) Normalized PL intensity of Yb^3+^ emission in CsPbCl_3_:Yb and CsPbCl_3_:Mn/Yb NCs at different temperature. e) PL spectra of CsPbCl_3_:Mn/Yb NCs different temperatures (297–477 K). Lifetime decay curves of f) exciton, g) Yb and h) Mn in CsPbCl_3_:Yb and CsPbCl_3_:Mn/Yb NCs at different temperatures.

**Figure** **7**a plots the PL spectra of CsPbCl_3_:Yb NCs at elevated temperatures from 297 to 477 K. Within the temperature range of 297 to 347 K, the Yb emission of CsPbCl_3_:Yb exhibits a slight increasing trend. We first analyzed the XRD at different temperatures and found that CsPbCl_3_:Yb does not undergo phase transitions, indicating that this abnormal optical property was not caused by a phase transition. Moreover, as the temperature increases, the Bragg diffraction peaks shift toward lower angles (Figure S18, Supporting Information). This shift is due to the lattice expansion caused by the increase in temperature. Additionally, we attribute the observed anomalous increase in PL to the desorption of water molecules with rising temperature.^[^ ^19^, ^50^ ^]^ Fourier Transform Infrared (FT‐IR) spectroscopy was employed to monitor the content of water molecules on the surface of NCs during the heating process (Figure S19, Supporting Information). The FT‐IR peak corresponding to the ‐OH vibrational group (≈3400 cm^−1^) shows a negative correlation with temperature, clearly indicating that water molecules desorb from the surface of NCs upon heating. Therefore, the observed negative thermal quenching of Yb is attributed to the higher temperature, which leads to the gradual desorption of water molecules from the surface of NCs, thereby reducing multi‐phonon relaxation. The PL lifetime of Yb^3+^ lengthens from 1.06 ms at 297 K to 1.4 ms at 387 K, which is also attributed to the disappearance of water molecules on the NCs’ surface due to the elevated temperature. This reduction in water molecules suppresses multi‐phonon decay, resulting in an extended decay lifetime (Figure 7c and Table S25, Supporting Information). Subsequently, we observed that in CsPbCl_3_:Yb, the exciton lifetime decreases as the temperature rises, which is due to the intensification of lattice vibrations, leading to non‐radiative transitions (Figure 7b and Table S26, Supporting Information).

The PL spectra of CsPbCl_3_:Mn/Yb NCs at high temperatures ranging from 297 to 477 K are shown in Figure 7e. Notably, the emission from the excitons and Mn^2+^ initially decreases, but the emission from Yb^3+^ significantly increases with the rise in temperature. Excitons and Yb have a similar trend in lifetime to that of CsPbCl_3_:Yb NCs (Figure 7f,g). As the temperature increases, the non‐radiative transition of excitons increases, leading to a decrease in lifetime (Table S27, Supporting Information). In contrast, FT‐IR detection revealed that as the temperature increases, the surface moisture of the NCs decreases (Figure S20, Supporting Information), initially increasing the Yb fluorescence lifetime. When the temperature becomes too high, it will also increase the non‐radiative transition of Yb, resulting in a decrease in lifetime (Table S28, Supporting Information). As shown in Figure S21 (Supporting Information), the XRD patterns at different temperatures are similar to those of CsPbCl_3_:Yb NCs. With the increase in temperature, exhibit a shift of Bragg diffraction peaks to lower angles due to lattice expansion. However, after this, the PL emission intensity of Yb still increased with the increase of temperature up to 427 K. By comparing the PL emission and lifetime of Mn at high temperatures between CsPbCl_3_:Mn and CsPbCl_3_:Mn/Yb, it was found that the PL emission and lifetime of Mn in CsPbCl_3_:Mn/Yb decreased more rapidly with increasing temperature than that of CsPbCl_3_:Mn (Figure 7h; Figure S22 and Table S29, Supporting Information). This illustrates the process of Mn‐Yb ET. The rate of ET can be defined as: Φ_Mn‐Yb_ = 1‐$\frac{\tau_{\mathrm{Mn}-\mathrm{Yb}}}{\tau_{\mathrm{Mn}}}$. Here, τ_Mn_ and τ_Mn‐Yb_ represent the average lifetimes of Mn and the Mn^2+^ emission lifetimes in CsPbCl_3_:Mn and CsPbCl_3_:Mn/Yb, respectively. Upon elevating the temperature from 297 to 477 K, the ET efficiency between Mn and Yb within the CsPbCl_3_ system improves significantly, rising from 9.5% to 45%. This proves that the transfer of Mn energy to Yb leads to negative thermal quenching of the PL emission of Yb. We attribute this phenomenon to lattice expansion caused by the increase in temperature, which leads to an increase in the distance between Mn‐Mn, reducing ET, and therefore enhancing the ET efficiency between Mn‐Yb.^[^ ^51^ ^]^ In addition, at the Mn level, the electrons in the excited level (^4^T_1_ _g_) pose a far higher risk to overpass the intersection point of ^4^T_1_ _g_ and ^6^A_1_ _g_ levels at high temperature with a subsequent non‐radiative transition to ground level due to the intensified thermal vibrations, thereby leading to the thermal quenching of Mn^2+^ emission. The doping of Yb^3+^ provides an extra multi‐channel for the thermally excited electrons migrating to the excited levels of Yb^3+^ ions. The electron supply from Mn^2+^ to Yb^3+^ ions compensates for the energy loss in Yb^3+^ emission caused by the increase in non‐radiative transitions at high temperatures, providing negative thermal quenching behavior (Figure S23, Supporting Information).^[^ ^52^ ^]^ When the temperature reaches 427 K, we observe that the emission of Mn is quenched, thus when the temperature rises to 477 K, the emission of Yb also appears to decline.^[^ ^52^ ^]^ By comparing the PL spectral intensity of CsPbCl_3_:Yb and CsPbCl_3_:Mn/Yb at different temperatures, it is found that CsPbCl_3_:Mn/Yb has better high‐temperature performance, and the emission of Yb reaches 3.2 times high at room temperature when the temperature is 427 K (Figure 7d). Table S30 (Supporting Information) summarizes the stability of near‐infrared luminescence of various fluorescent materials. This work positions the fluorescent material in a relatively stable location compared to previous ones, indicating the potential commercial value of CsPbCl_3_:Mn/Yb NCs.

**Figure 8:**
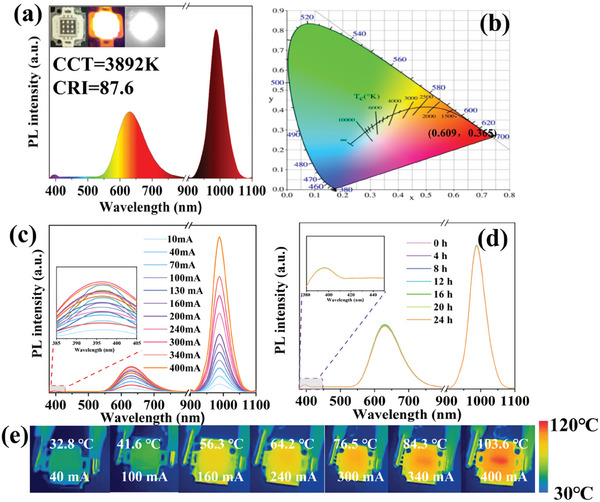
Performance of pc‐LED device based on CsPbCl_3_:Mn/Yb NCs. a) EL spectra, Insets presents photographs of the UV chip and the constructed pc‐LED device. b) CIE color coordinates. The EL spectra of the pc‐LED device operating at different c) currents and d) time intervals. e) The actual temperature variations of the pc‐LED device under different forward bias currents (with a fixed voltage of 10 V).

In order to further explore the potential applications of CsPbCl_3_:Mn/Yb NCs in Vis‐NIR‐LED devices, we have investigated their thermal stability and operational lifetime performance. The synthesized NCs@polydimethyl siloxane (NCs@PDMS) thin film was integrated with a commercial UV chip at 365 nm to fabricate a pc‐Vis‐NIR‐LED device. This LED emits orange light that is perceptible to the human eye, while the NIR camera can detect intense NIR light (inset of **Figure** **8**a). As shown in Figure 8a, the emission peak for the orange light provided by Mn is located at 630 nm, and the emission peak for the near‐infrared light provided by Yb is situated at 980 nm. These peaks correspond well with those of the CsPbCl_3_:Mn/Yb NCs. The LED device offers a bright orange emission with color coordinates of (0.609, 0.365) (Figure 8b), a correlated color temperature (CCT) of 3892 K, and a color rendering index (CRI) of 87.6. Furthermore, the electroluminescence (EL) spectra, as depicted in Figure 8c, reveal a progressive enhancement of both the NIR emissions from the pc‐Vis‐NIR‐LED with an increase in the driving current from 10 to 400 mA, confirming the NIR emission shows no significant saturation effect in response to UV light. However, the emission from excitons and Mn exhibits a saturation‐like phenomenon, which may be due to the fact that as the current increases, the commercial UV chips may generate heat, affecting their emissions. As shown in Figure 8d, the photostability at 100 mA was tested at different time intervals, and no significant degradation or changes were detected over 24 h. This confirms that the pc‐Vis‐NIR‐LED constructed with NCs@PDMS possesses superior EL stability. It is well known that an increase in forward current correlates with a rise in chip temperature, which can induce thermal quenching in the emissive NCs. To further investigate this, real‐time infrared thermography technology was utilized to monitor the temperature of commercial chips emitting at 365 nm UV light at different currents (Figure 8e). As the input current increases, the actual temperature of the UV chip also rises. At a current of 400 mA, the temperature rises to 103.6 °C. However, the optical performance of CsPbCl_3_:Mn/Yb remains essentially unaffected. The pc‐Vis‐NIR‐LED can operate normally at this temperature, suggesting that this LED device can function effectively even under conditions where heat is generated by the increased current.

**Figure 9:**
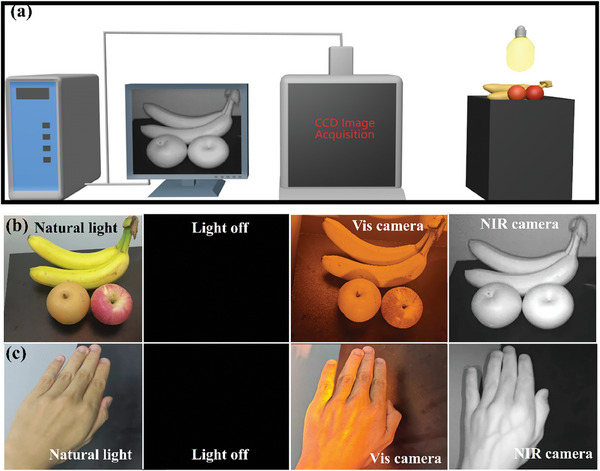
a) Schematic diagrams for the application of as‐fabricated pc‐Vis‐NIR‐LED device. Photographs of b) fruits and c) palm taken with a visible camera and NIR camera when the as‐fabricated pc‐Vis‐NIR‐LED device is turn on or turn off, respectively.

As depicted in **Figure** **9**a, an NIR camera and image processing system are employed to achieve the collection and processing of items illuminated by the pc‐Vis‐NIR‐LED devices. A charge‐coupled device (CCD) image sensor is connected to a personal computer, serving as the image acquisition unit. The CCD image sensor captures images of items illuminated by the pc‐Vis‐NIR‐LED devices in a dark environment upon the application of electrical power. Subsequently, computer software is utilized to process the acquired images, functioning as the signal processing unit. This processing step results in the mapping information of the items.

Figure 9b,c present photographs of a fruit and a palm taken under natural light and pc‐Vis‐NIR‐LED light, respectively. When the pc‐Vis‐NIR‐LED is turned off, the NIR camera and Vis camera fail to capture an image. In contrast, when the LED device is active, the visible camera captures an orange image, and the infrared camera captures a black‐and‐white image, demonstrating the dual‐emission capabilities of the pc‐Vis‐NIR‐LED. These findings distinctly highlight the potential applications of CsPbCl_3_:Mn/Yb NCs in orange light illumination and night vision technology. Beyond these applications, the versatility of this type of phosphor extends to other promising fields, such as the food and medical industries, where its unique properties are anticipated to hold significant value.

## 3 Conclusion

In summary, we have successfully devised an innovative and adaptable strategy for the synthesis of CsPbCl_3_:Mn/Yb NCs. This method utilizes the photoinduced cleavage of the covalent CCl_4_ bond in haloalkanes to provide chloride ions to achieve the regulation of CsPbCl_3_:Mn/Yb NCs, offering a significant advancement over traditional synthesis techniques such as hot‐injection and supersaturated recrystallization. Furthermore, our research has revealed a significant enhancement in the high‐temperature performance of Yb when Yb and Mn are co‐doped into CsPbCl_3_ NCs. At temperature of 427 K, the PL intensity of Yb is observed to increase by a factor of 3.2 compared to its initial state. This substantial enhancement in robustness can be ultimately ascribed to the synergistic energy interchange between Mn and Yb, coupled with a notable reduction in the surface moisture of CsPbCl_3_:Mn/Yb NCs. The CsPbCl_3_:Mn/Yb‐integrated pc‐Vis‐NIR‐LED exhibits vibrant PL with peak emissions at ≈600 and 1000 nm with a light excitation of 365 nm commercial UV LED. The pc‐Vis‐NIR‐LED has superior light stability without significant degradation after operating at a current of 100 mA for up to 24 h. Such characteristics position this pc‐Vis‐NIR‐LED as a versatile solution for applications involving both visible light and near‐infrared illumination, offering a promising avenue for advancements in lighting technology and beyond.

## 4 Experimental Section

### Chemicals

Lead acetate (Pb(CH_3_COO)_2_·3H_2_O, 99.5%), cesium carbonate (Cs_2_CO_3_,99%), manganese acetate (Mn(CH_3_COO)_2_·4H_2_O, 98%), ytterbium acetate (Yb(CH_3_COO)_2_·4H_2_O, 98%) were purchased from Aladdin (Shanghai, China). Oleic acid (OA, 90%), acetone (anhydrous, 99.8%), oleylamine (OLA, 90%), and 1‐octadecene (ODE, 90%) were purchased from Sigma–Aldrich (Shanghai, China). Polydimethylsiloxane (PDMS, 99%), Cyclohexane, ethanol, and tetrachloromethane (CCl_4_, 98%) were purchased from Sinopharm Chemical Reagent Co. (Shanghai, China). All the chemical reagents were used as received without further purification, and distilled water was used throughout the experiments.

### Preparation of Cs, Pb, Mn, and Yb Oleate Precursors

4 mmol Cs_2_CO_3_, 4 mmol Pb(CH_3_COO)_2_·3H_2_O, 4 mmol Mn(CH_3_COO)_2_·4H_2_O, 4 mmol Yb(CH_3_COO)_2_·4H_2_O were mixed with 4 mL OA, 4 mL OLA and 12 mL ODE in a 50 mL three‐neck flask, respectively. The resulting mixture was heated to 120 °C under N_2_ atmosphere with constant stirring for 30 mins to remove water and oxygen, and then heated to 160 °C and stirred for 1 h to form a clear solution. For the preparation of Pb‐ and Mn‐oleate precursors, all the conditions were the same as that of Cs‐oleate precursor, except that Pb(CH_3_COO)_2_·3H_2_O and Mn(CH_3_COO)_2_·4H_2_O were respectively used instead of Cs_2_CO_3_.

### Photoinduced Synthesis of CsPbCl_3_, CsPbCl_3_:Mn, CsPbCl_3_:Yb, CsPbCl_3_:Mn/Yb NCs

Briefly, 10 µL stock solution of Pb‐oleate and 10 µL stock solution of Cs‐oleate were dispersed in 3 mL of CCl_4_ at room temperature, CsPbCl_3_ was prepared by dispersing 10 µL of Pb‐oleate and 10 µL of Cs‐oleate in 3 mL of carbon tetrachloride and then subjected to photoirradiation with a 365 nm UV LED (The 365 nm LED should utilize a broad‐spectrum light to ensure complete coverage of the cuvette, in order to prevent uneven reactions within the solution in the cuvette). The preparation method of CsPbCl_3_:Mn, CsPbCl_3_:Yb, CsPbCl_3_:Mn/Yb NCs were the same as that of CsPbCl_3_, except that Mn‐OA or Yb‐OA was added. This method allows control over the growth rate and nucleation by adjusting the light intensity and exposure time. The crude solution was centrifuged at 12 000 rpm for 5 min to collect the NCs. The precipitate was then dispersed in 2 mL of cyclohexane and centrifuged again at 12 000 rpm for 5 min. After centrifugation, the supernatant was discarded, and the NCs were redispersed in 2 mL of cyclohexane.

### Construction of CsPbCl_3_:Mn/Yb Visible‐NIR LED Device

The device was constructed by directly coupling the as‐fabricated CsPbCl_3_:Mn/Yb film on the 365 nm commercial UV LED chip. The CsPbCl_3_:Mn/Yb NCs were homogeneously dispersed in a mixture of A/B gels (1:1, k‐9761, Kafuter, Guangzhou Hengda New Material Co., Ltd) and then dropped on the chip. The major component of the A gel was epoxy resin, and the B gel contained the curing agent. Additionally, opaque silica gels were filled around the edges of the device to prevent the leakage of UV light. The emitting area of pc‐NIR‐LED was 0.06 cm^2^.

### Characterization

Powder X‐ray diffraction patterns (XRD) of the samples were collected on an X‐ray diffractometer (MiniFlex 600, Rigaku) with Cu Kα_1_ radiation (λ = 0.154187 nm). Both the low and high‐resolution transmission electron microscopy (TEM) measurements were performed by using a TECNAI G2 F20 TEM equipped with the energy‐dispersive X‐ray (EDX) spectrum. The X‐ray photoelectron spectroscopy (XPS) measurement for CsPbCl_3_, CsPbCl_3_:Mn, CsPbCl_3_:Yb, and CsPbCl_3_:Mn/Yb NCs were conducted on an Ulvac PHI/X‐tool spectrometer with Al K_ɑ_ radiation source (1486.6 eV, 24.1 W, 15 kV) and a beam diameter of 100 × 100 µm. UV–vis absorption spectra of samples were collected by a Perkin–Elmer Lambda 365 UV/Vis spectrometer in transmission mode. PL excitation spectra, PL emission spectra, and PL decays were recorded on an FLS1000 spectrometer (Edinburgh) equipped with both continuous xenon (450 W) and pulsed flash lamps. The PLQY of CsPbCl_3_, CsPbCl_3_:Mn, CsPbCl_3_:Yb, and CsPbCl_3_:Mn/Yb NCs were obtained by employing a standard barium sulfate‐coated integrating sphere (150 nm in diameter, Edinburgh) as the sample chamber that was mounted on the FLS1000 spectrometer with the entry and output port of the sphere located in 90° geometry from each other in the plane of the spectrometer. FT‐IR spectra were collected in a Nicolet 380 FTIR infrared spectrometer. Note that the sample was treated with an infrared lamp for 10 min before the heating FT‐IR measurement.

## Conflict of Interest

The authors declare no conflict of interest.

## Supporting information



Supporting Information

## Data Availability

The data that support the findings of this study are available from the corresponding author upon reasonable request.
