# Supplementary material for: Photo‐Induced Synthesis of Ytterbium and Manganese‐Doped CsPbCl3 Nanocrystals for Visible to Near‐Infrared Photoluminescence with Negative Thermal Quenching
Source: Adv Sci (Weinh). 2024 Dec 4;12(4):2408927. doi: 10.1002/advs.202408927 (PMC11775517; doi:10.1002/advs.202408927)
Supplement: Supplementary file 1 — Supporting Information [file ADVS-12-2408927-s001.docx]

Supporting Information

Photo-induced Synthesis of Ytterbium and Manganese-Doped CsPbCl_3_ Nanocrystals for Visible to Near-Infrared Photoluminescence with Negative Thermal Quenching

Xiaochen Fang*^a,+^*, Zhuo Chen*^b,+^*, Mandy Hei Man Leung*^c^*, Biao Zheng*^d,*^*, Liwei Wang*^d,*^*, Meng An*^b,*^*, Yusuke Asakura*^c,^*,* Yusuke Yamauchi*^c,e^* and Zhanhui Yuan*^a,*^*

*^a^*College of Materials Engineering, Fujian Agriculture and Forestry University, Fuzhou 350002, China

*^b^*College of Mechanical and Electrical Engineering, Shaanxi University of Science and Technology, Xi’an 710021. P. R. China

*^c^*Department of Materials Process Engineering, Graduate School of Engineering, Nagoya University, Nagoya 464-8603, Japan.

*^d^*Fujian Key Laboratory of Functional Marine Sensing Materials, College of Material and Chemical Engineering, Minjiang University, Fuzhou 350108, China

*^e^*School of Chemical Engineering and Australian Institute for Bioengineering and Nanotechnology (AIBN), The University of Queensland, Brisbane, QLD 4072, Australia.

^+^ Xiaochen Fang and Zhuo Chen contributed equally.

Computational Method

The first principles calculations were performed using density function theory (DFT) as implemented in the Vienna ab initio simulation package (VASP).^1^ The projector augmented wave (PAW) method is used to describe the interaction between ionic cores and valence electrons.^2^ The exchange-correlation functional is treated through the generalized gradient approximation (GGA) in the form of Perdew−Burke−Ernzerhof (PBE-sol)_._^3, 4^ The cutoff energy of the plane-wave basis set is 500 eV. The lattice parameters of unit cell for CsPbCl_3_ are firstly optimized, and *a* = *b* = *c* = 5.53 Å. Then, a 2×2×2 supercell with 40 atoms is relaxed and used for the calculations of energy band and partial density of states (PDOS). The surface Brillouin zones are sampled using a Γ-centered Monkhorst-Pack. The 2×2×2 and 3×3×3 k-point mesh are used for geometry optimization and electronic property calculations, respectively. The self-consistent iterative loop convergence criterion is 10^-6^ eV in electronic relaxation and all the atoms are fully relaxed until the force on each atom is less than 0.02 eV/Å. Besides, we consider the spin polarised calculation in CsPbCl_3_:Mn and CsPbCl_3_:Mn/Yb.


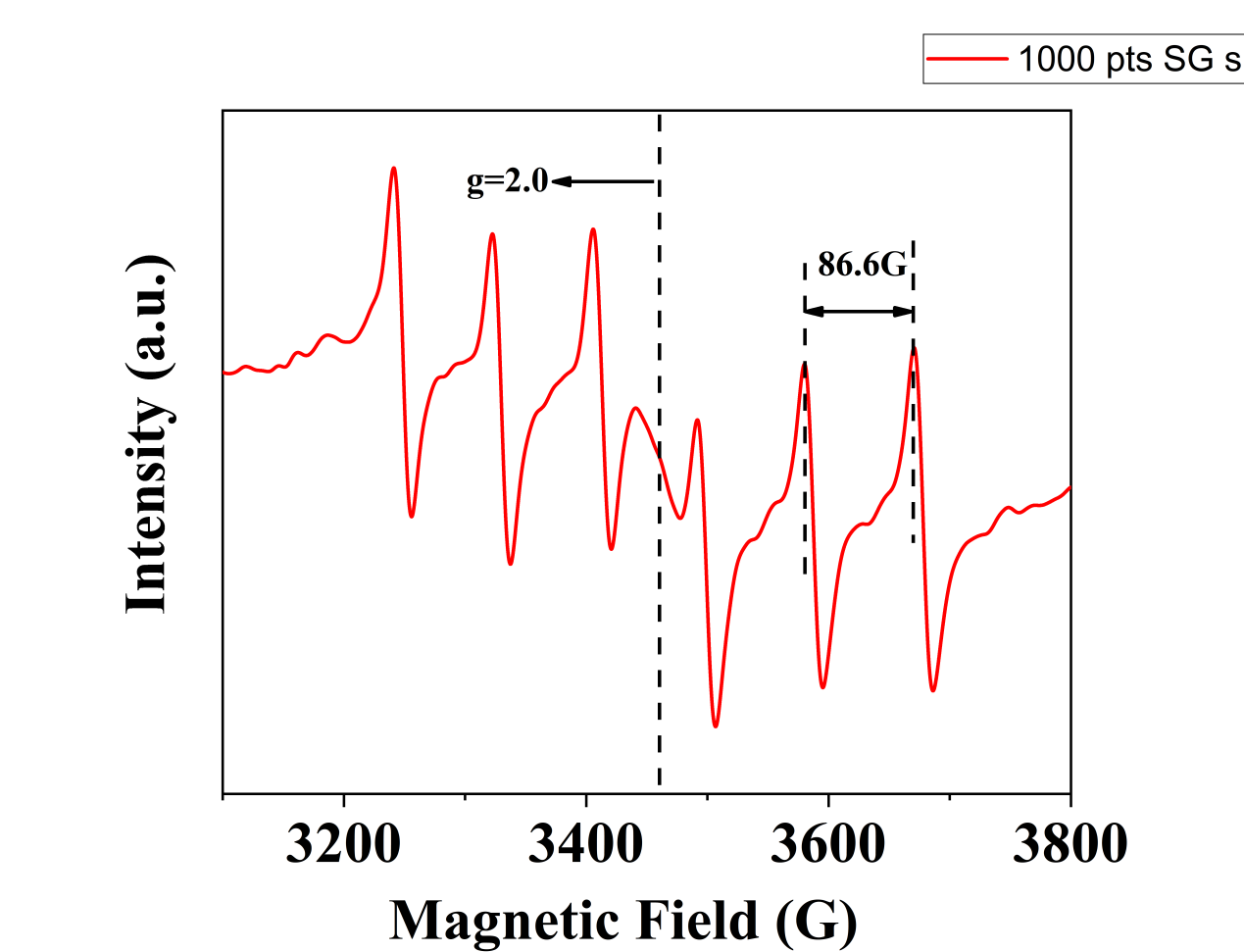


**Figure S1.** The EPR spectrum of CsPbCl_3_:Mn/Yb NCs at room temperature.


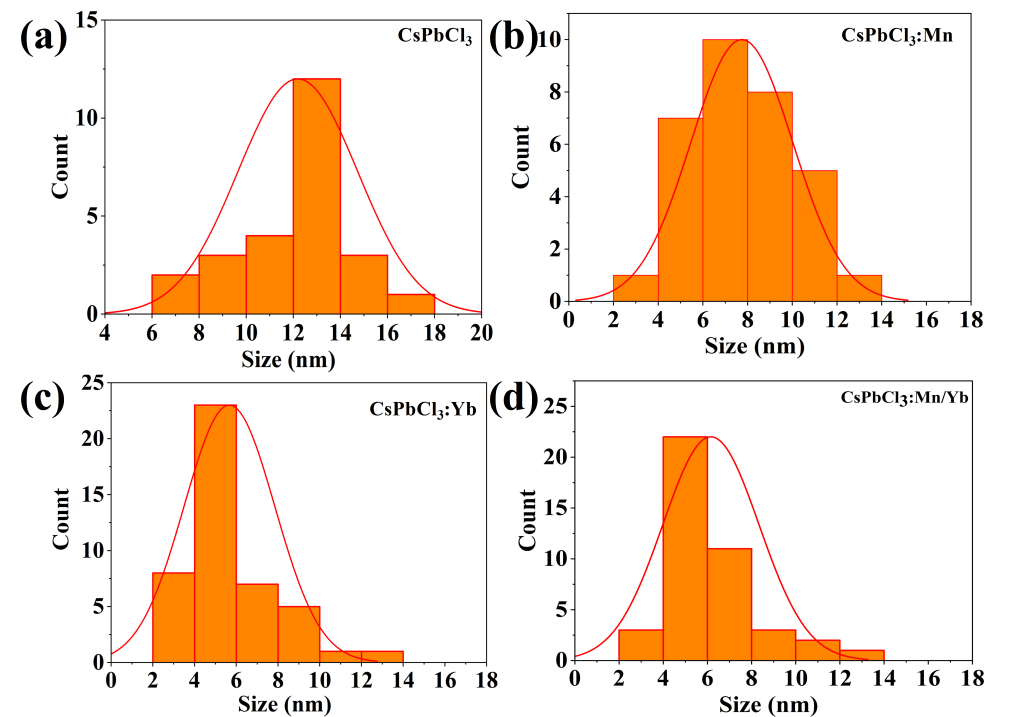


**Figure S2.** Size histograms for the undoped, Mn-doped, Yb-doped, and Mn/Yb-doped CsPbCl_3_ NCs.


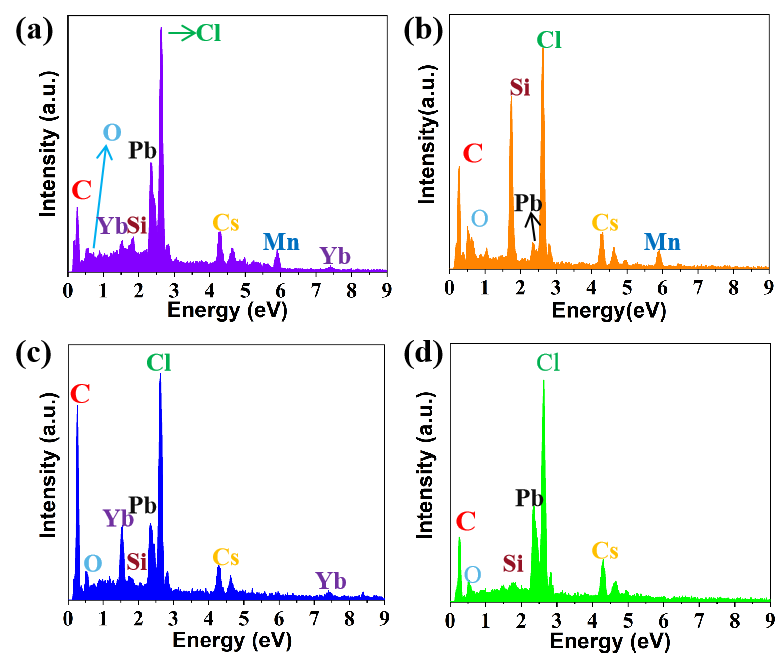


**Figure S3.** EDX spectra of (a) undoped, (b) Mn-doped, (c) Yb-doped, and (d) Mn/Yb-doped CsPbCl_3_ NCs.


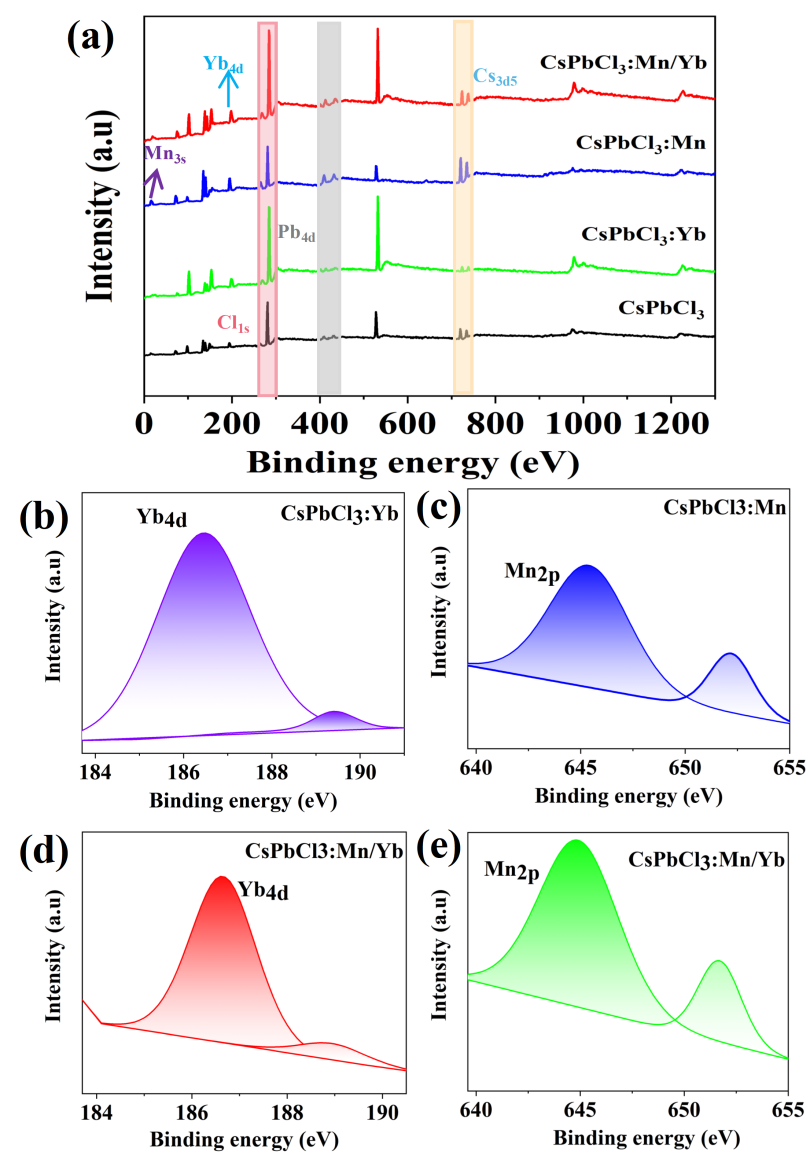


**Figure S4.** (a) Wide-scan XPS spectra of undoped, Mn-doped, Yb-doped, and Mn/Yb-doped CsPbCl_3_ NCs. Narrow-scan XPS spectra for Yb:4d in (b) Yb-doped and (d) Mn/Yb-doped CsPbCl_3_ NCs. Narrow-scan XPS patterns for Mn:2p in (c) Mn-doped and (e) Mn/Yb-doped CsPbCl_3_ NCs.


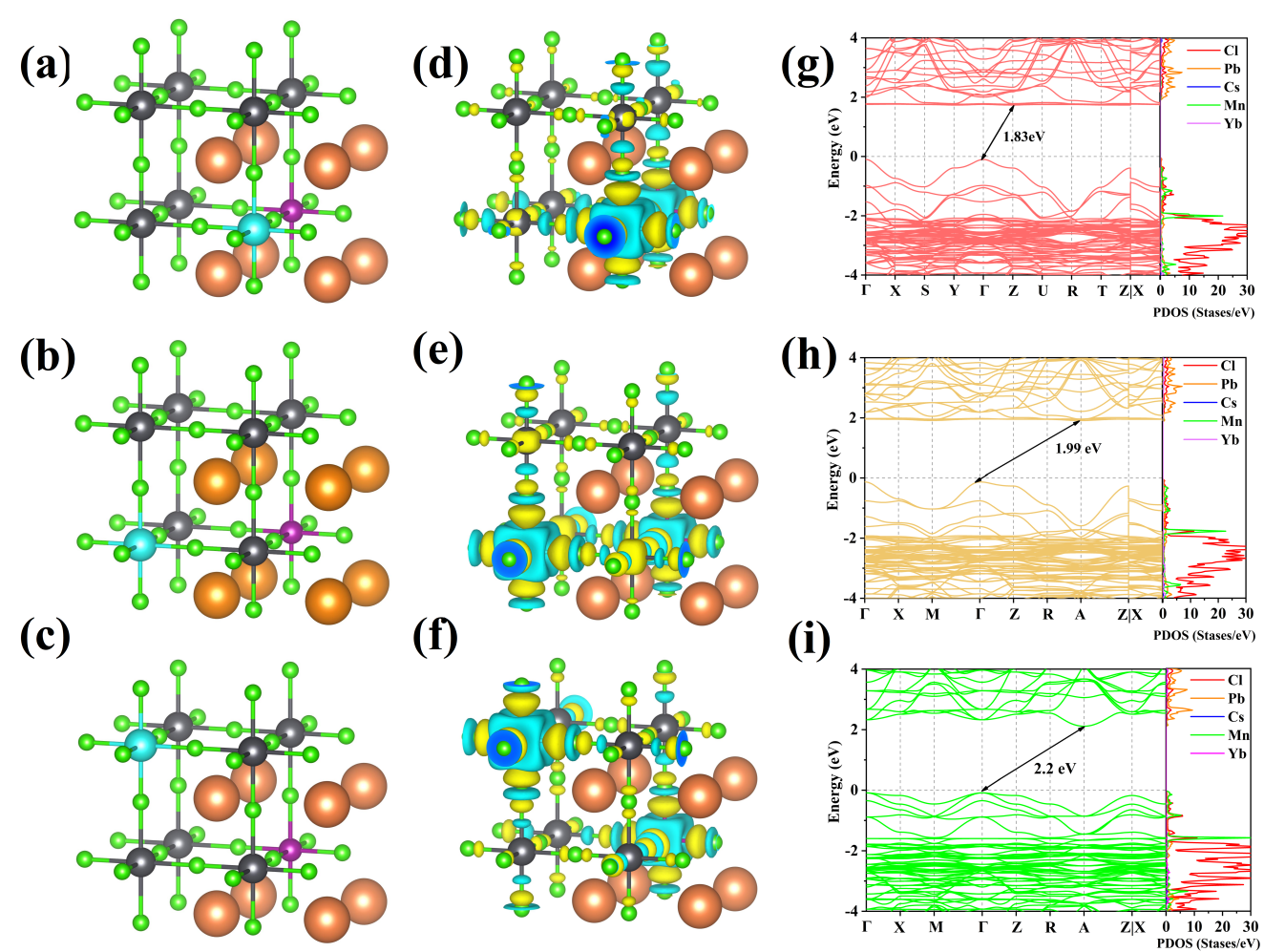


**Figure S5.** (a-c) The lattice structures, (d-f) calculated deformation charge densities, and (g-i) band structures of Mn and Yb at different positions in CsPbCl_3_ lattice.


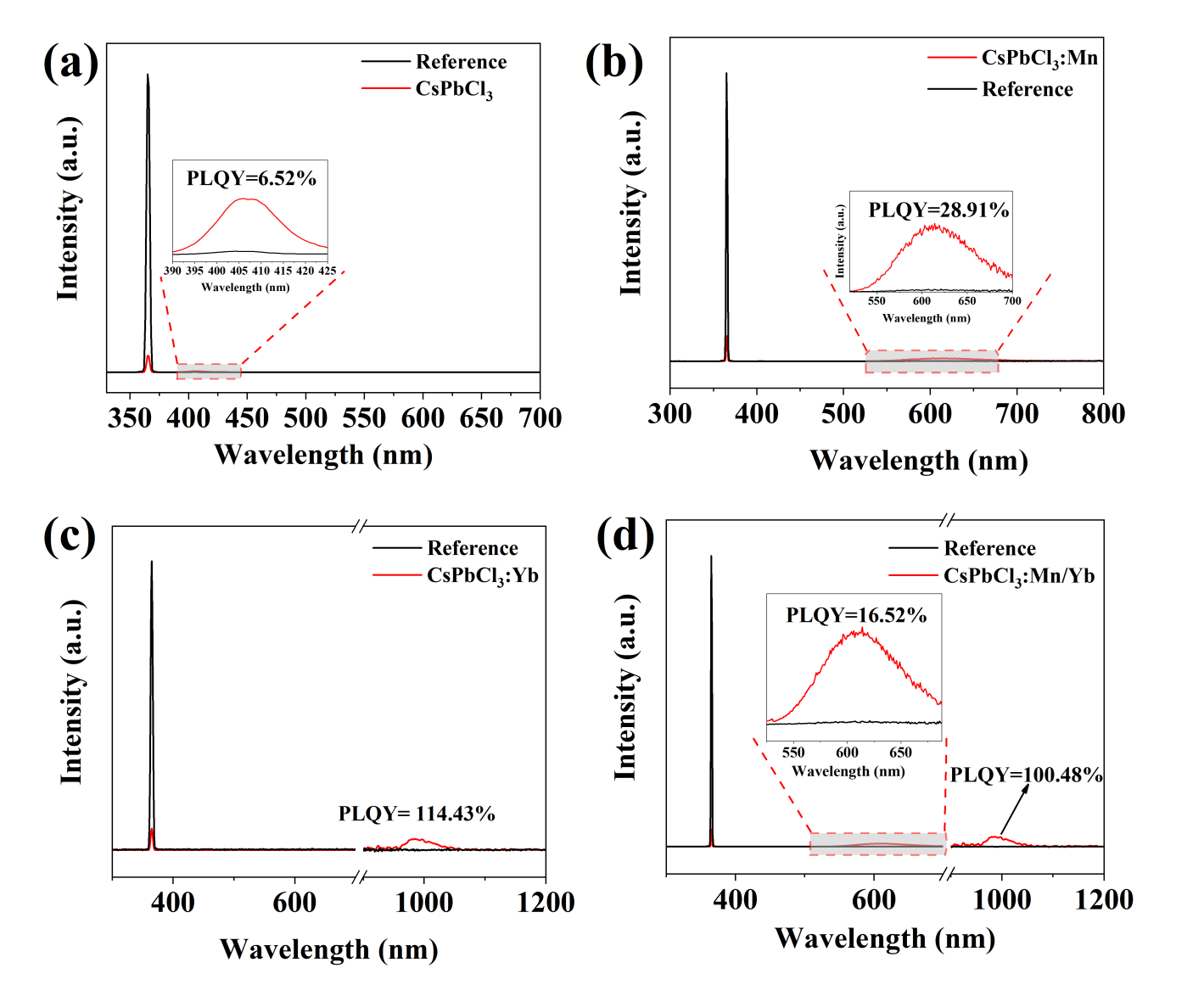


**Figure S6.** The photoluminescence (PL) spectra of (a) CsPbCl_3_, (b) CsPbCl_3_:Mn, (c) CsPbCl_3_:Yb, and (d) CsPbCl_3_:Mn/Yb samples were recorded for the determination of PLQY using a spectrofluorometer equipped with an integrating sphere.


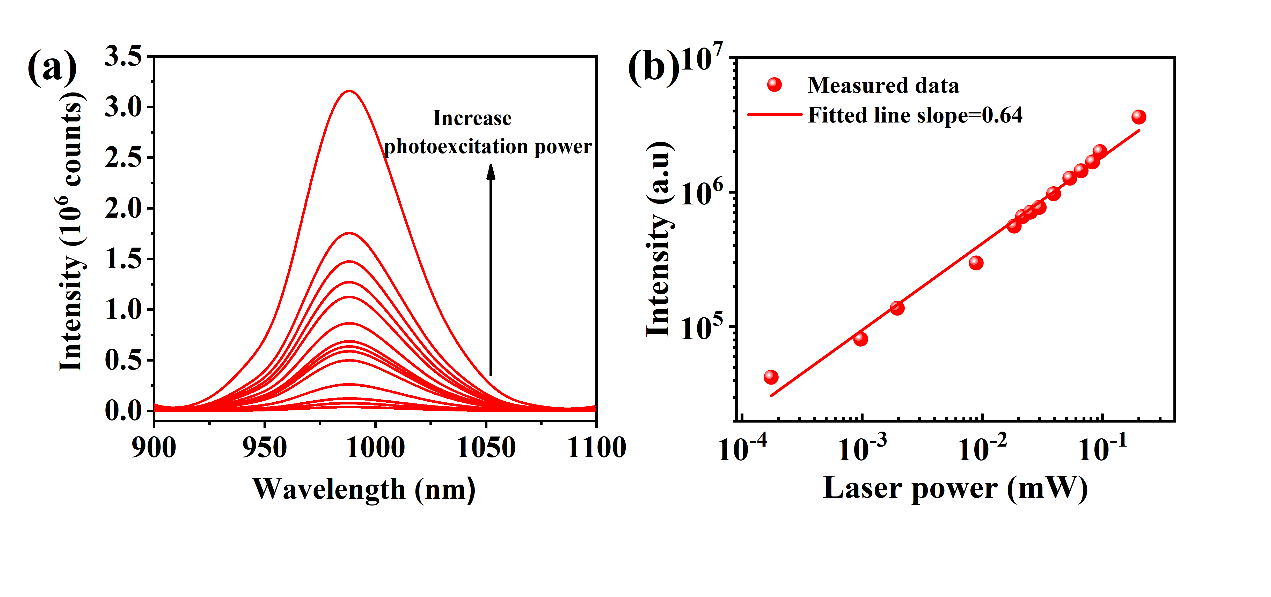


**Figure S7.** (a) Room-temperature PL spectra of the CsPbCl_3_:Mn/Yb NCs collected as a function of increasing photoexcitation power from a 375 nm laser diode. (b) the log−log plot of NIR emission intensity versus photoexcitation power.


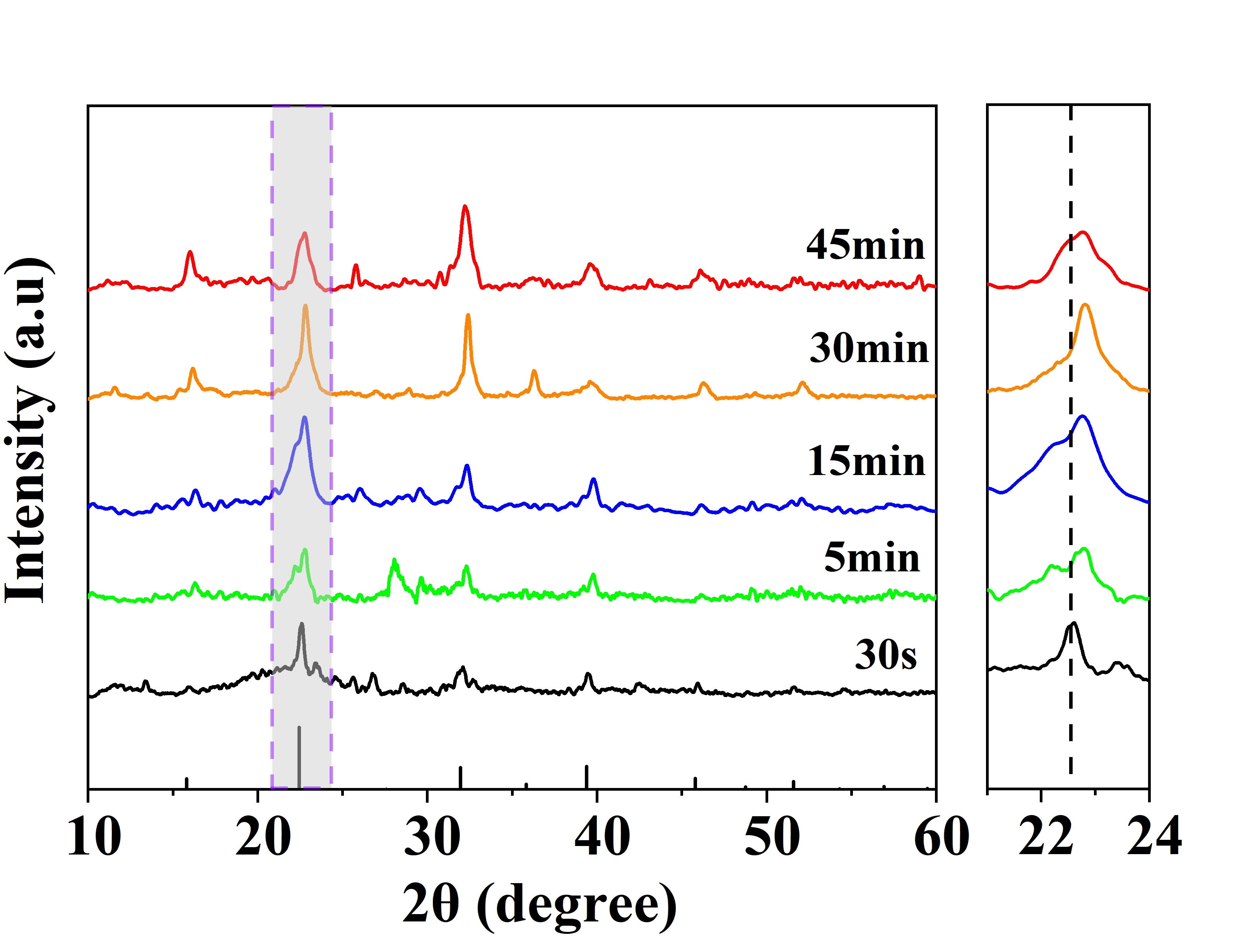


**Figure S8**. XRD patterns of Mn/Yb-doped CsPbCl_3_ NCs prepared with different UV irradiation times.


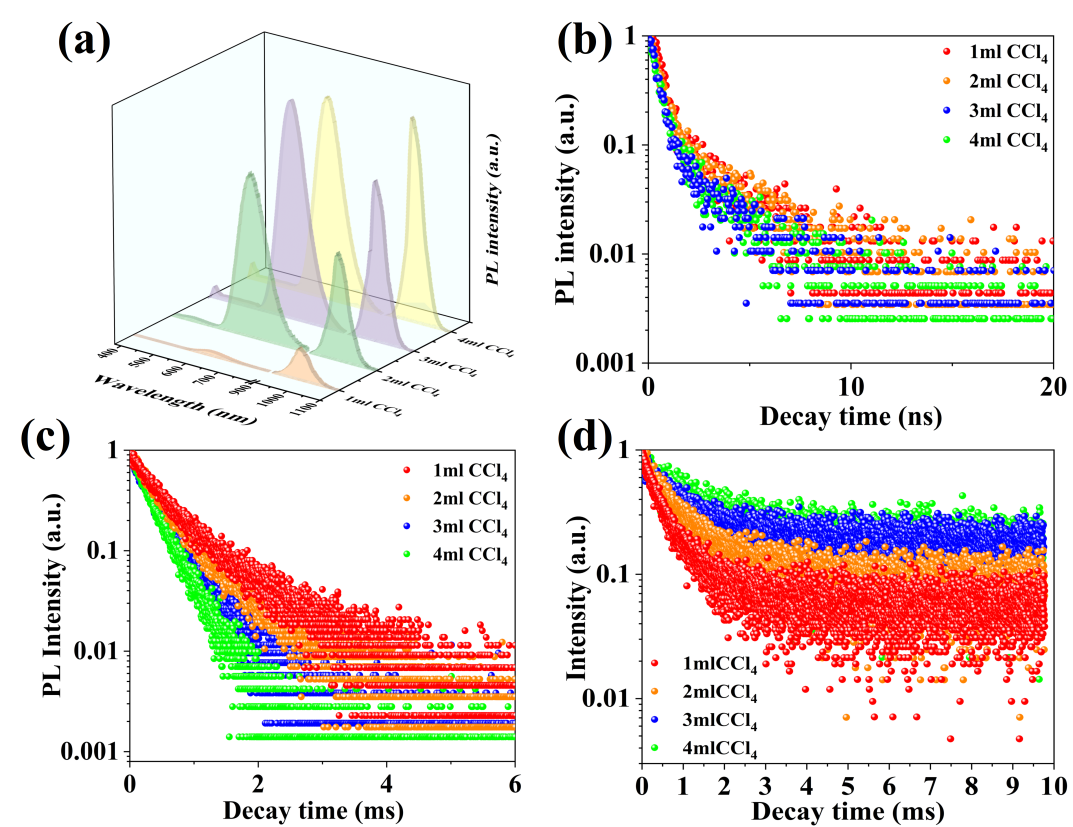


**Figure S9.** (a) PL spectra of CsPbCl_3_:Mn/YbNCs prepared with different CCl_4_ contents. Changes in the lifetimes of (b) exciton recombination, (c) Mn emission, and (d) Yb emission in CsPbCl_3_:Mn/YbNCs prepared with different CCl_4_ contents.


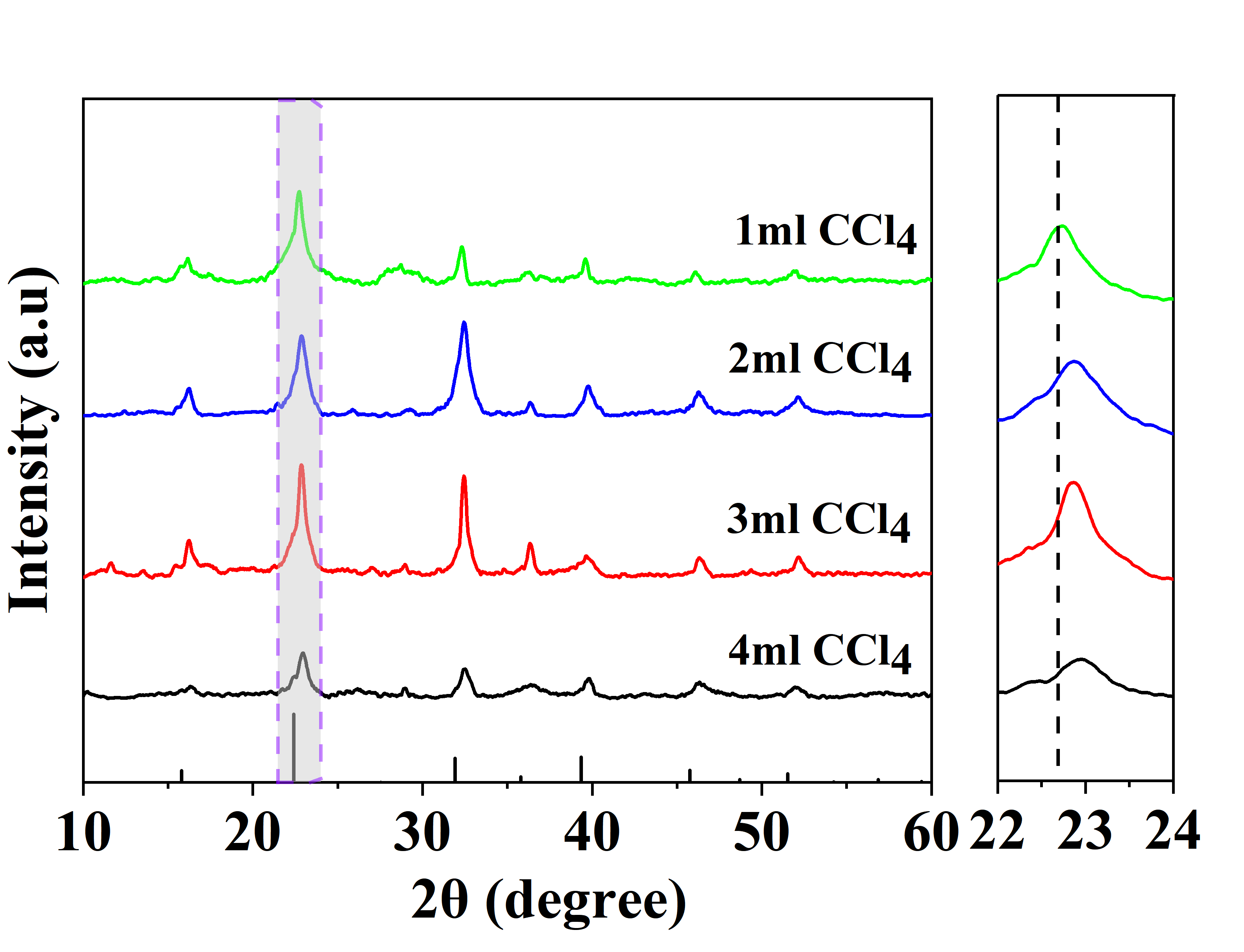


**Figure S10.** XRD patterns of Mn/Yb-doped CsPbCl_3_ NCs prepared with different CCl_4_ contents.


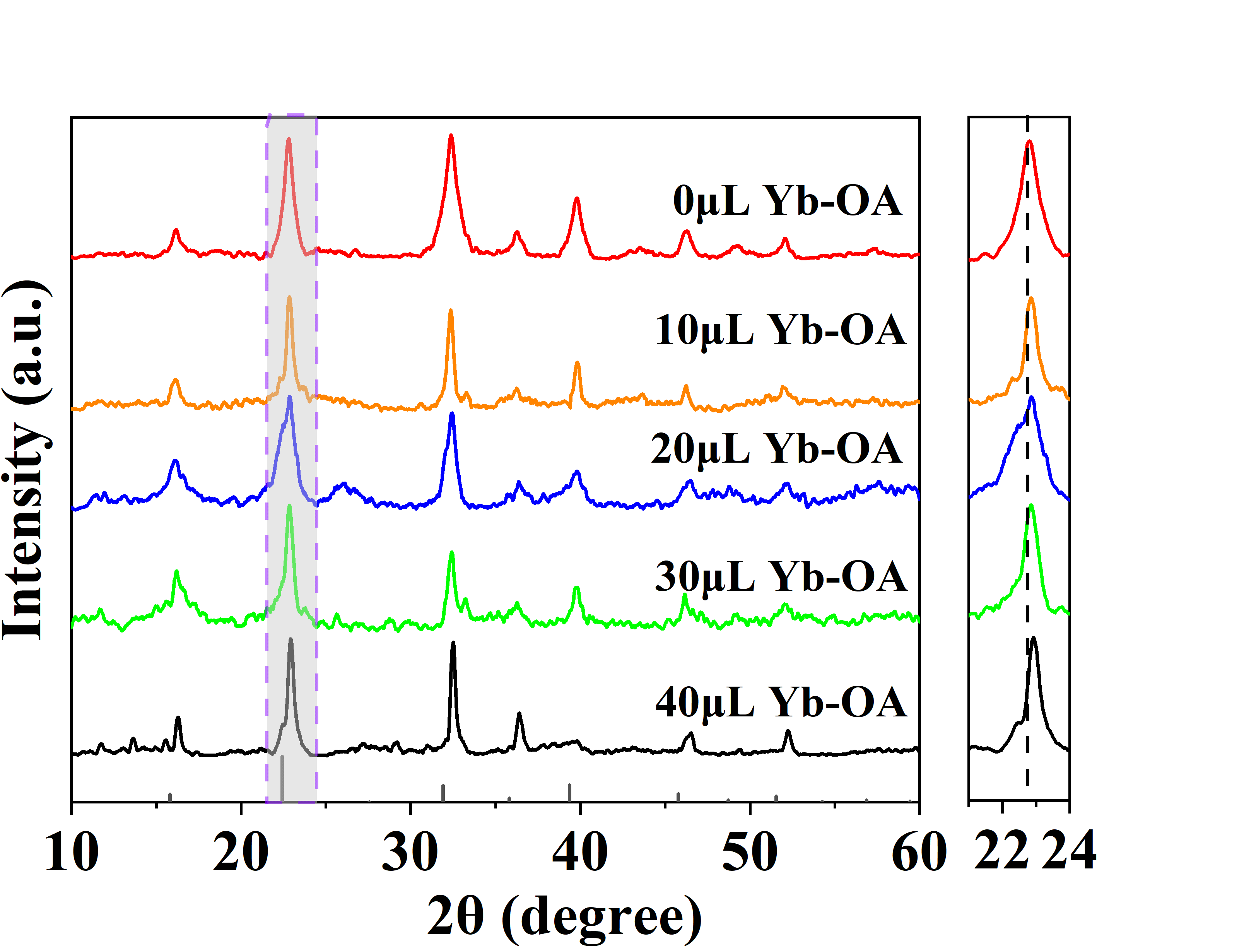


**Figure S11.** XRD patterns of Mn/Yb-doped CsPbCl_3_ NCs prepared with different Yb-OA contents.


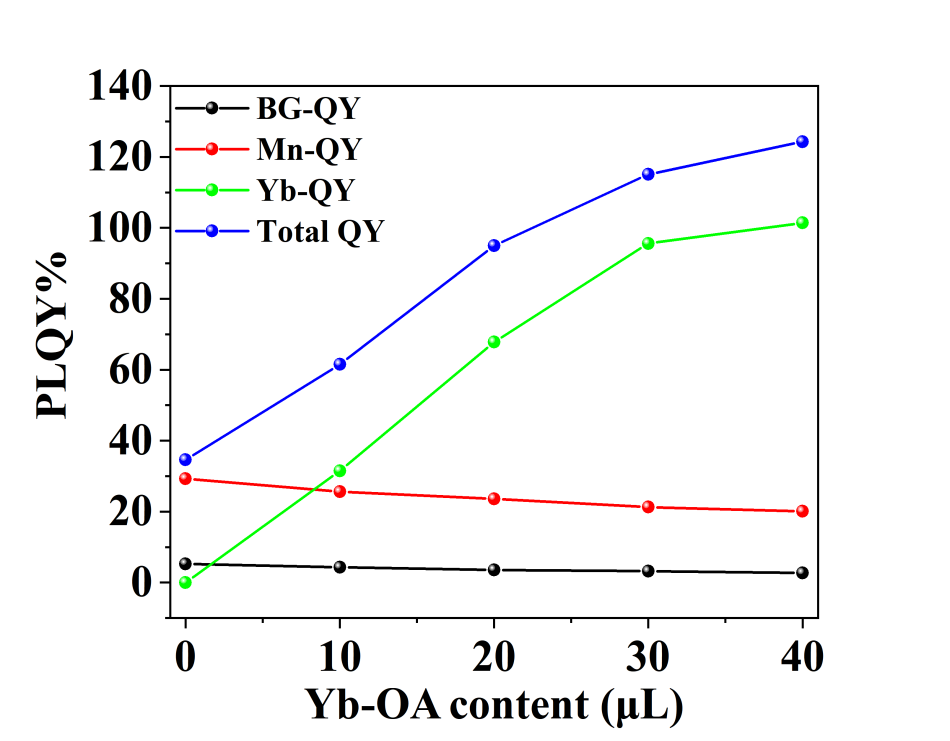


**Figure S12.** PLQY of CsPbCl_3_:Mn/Yb NCs prepared with different Yb-OA contents.


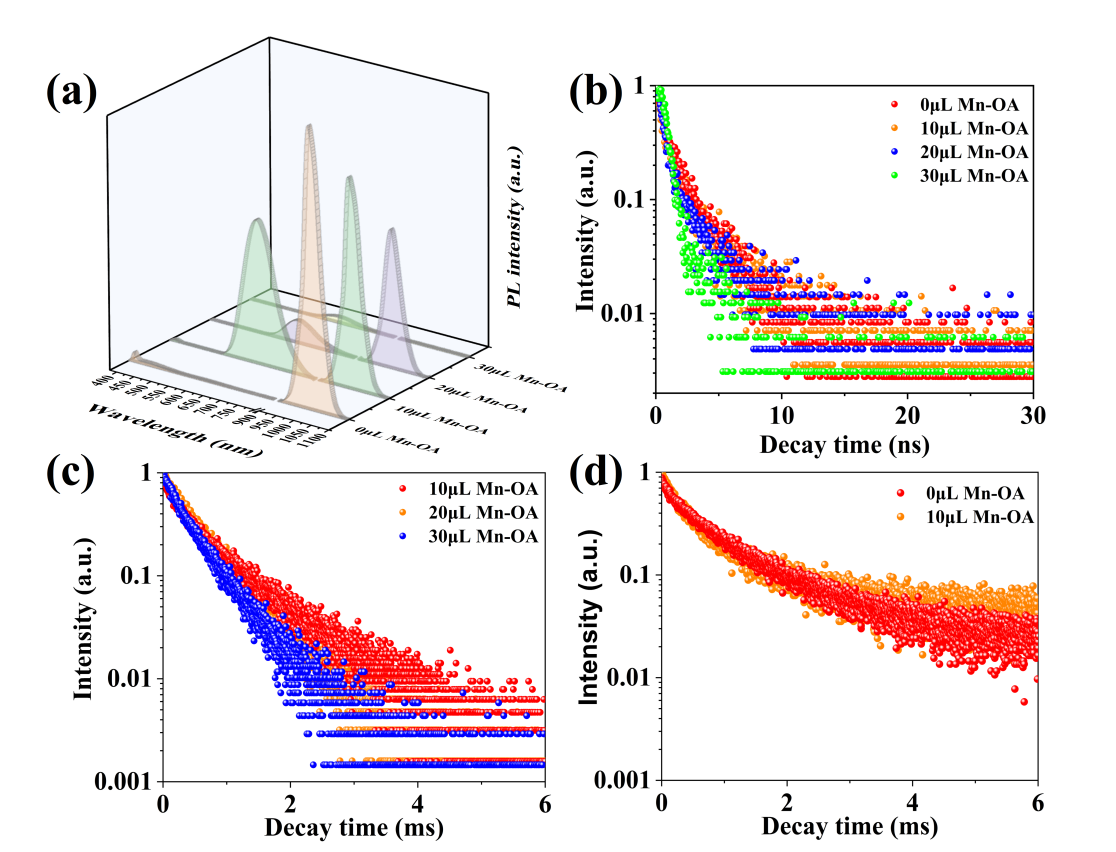


**Figure S13.** (a) PL spectra of CsPbCl_3_:Mn/Yb NCs prepared with different Mn-OA contents. Changes in the lifetimes of (b) exciton recombination, (c) Mn emission, (d) Yb emission in CsPbCl_3_:Mn/Yb NCs prepared with different Mn-OA contents.


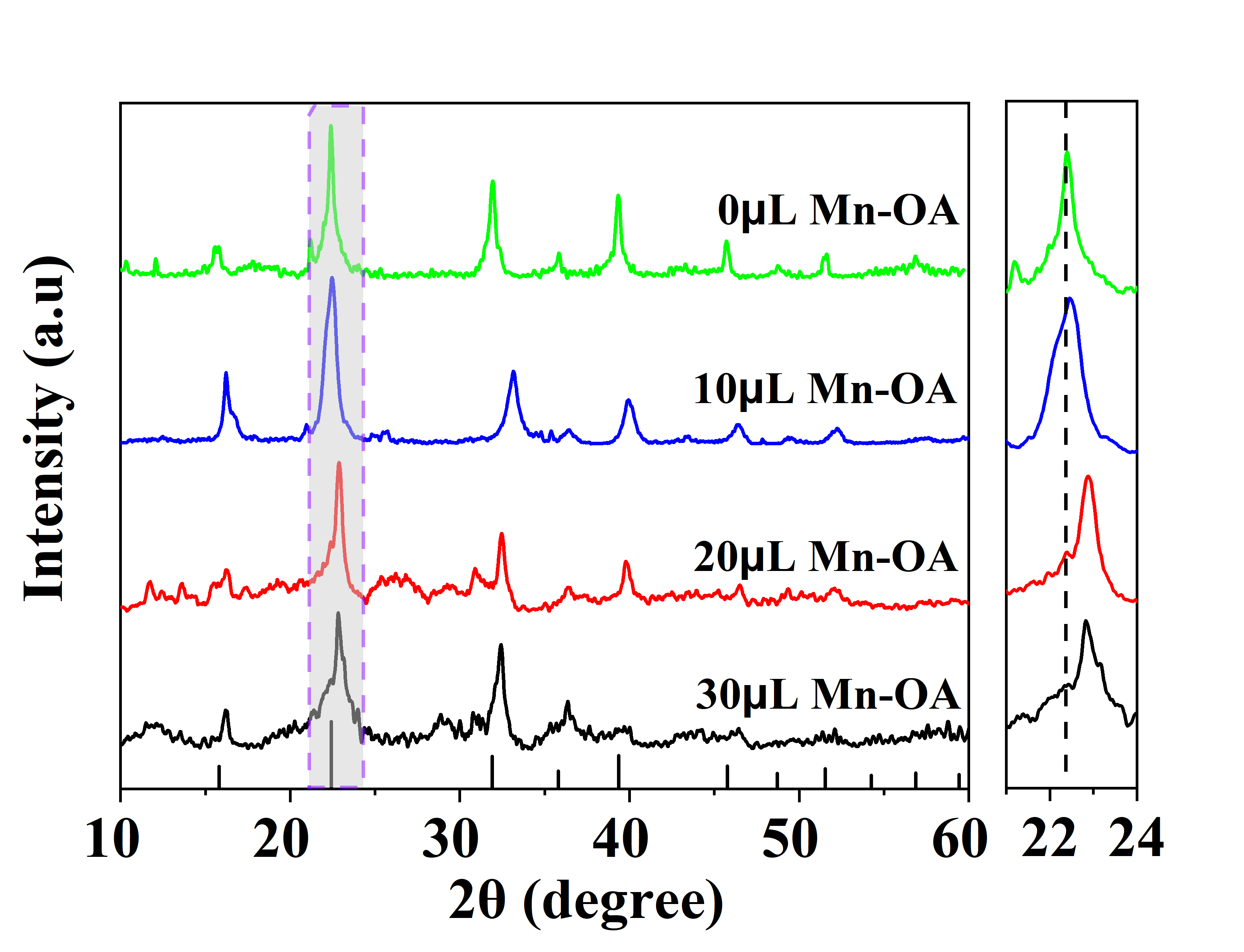


**Figure S14.** XRD patterns of Mn/Yb-doped CsPbCl_3_ NCs prepared with different Mn-OA contents.


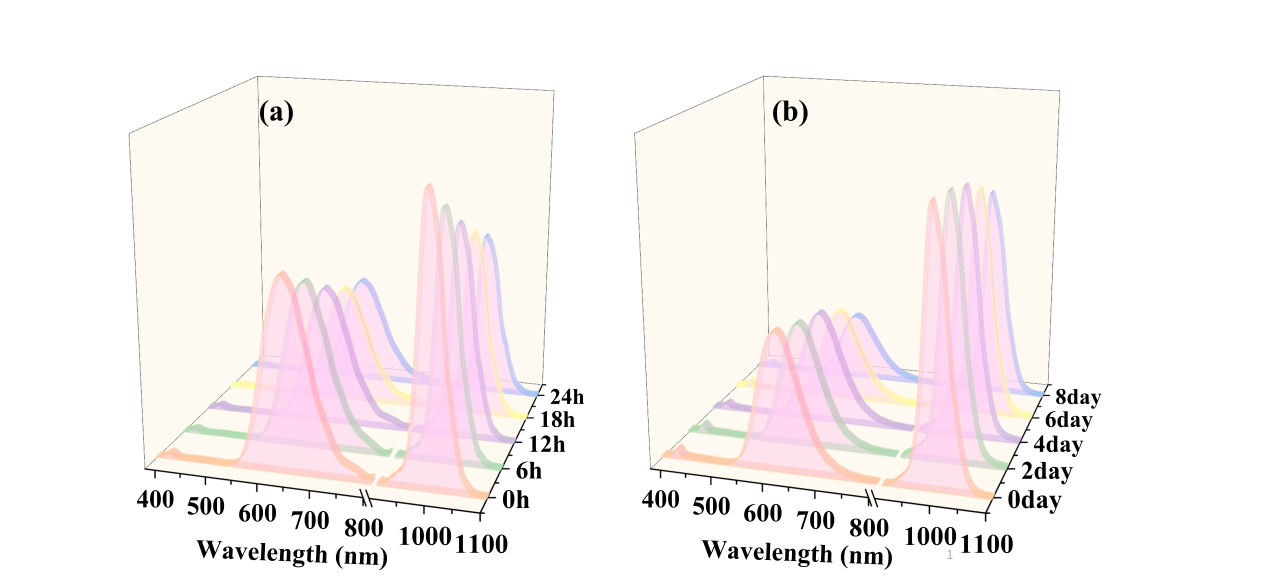


**Figure S15.** The PL spectra of CsPbCl_3_:Mn/Yb NCs under different (a) UV irradiation times and (b) storage times.


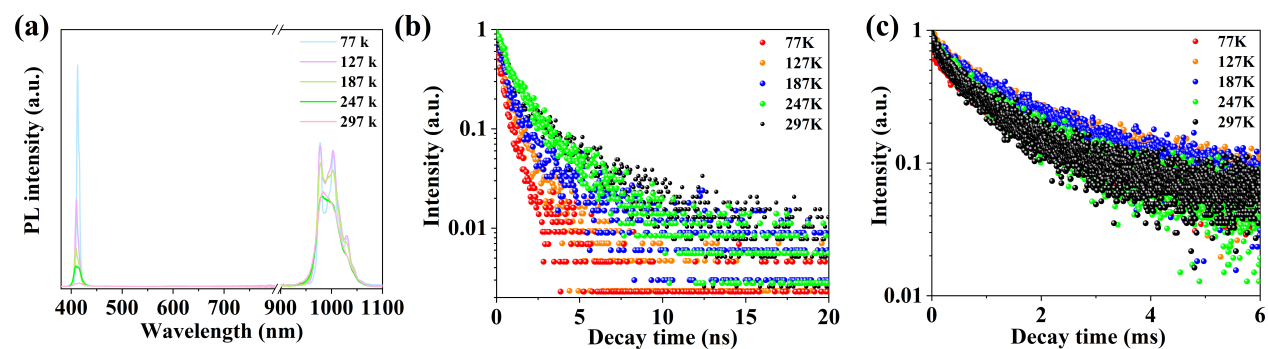


**Figure S16.** (a) Temperature-dependent (77 K-297 K) steady-state PL spectra of CsPbCl_3_:Yb NCs. Changes in the lifetimes of (b) exciton recombination and (c) Yb emission under different temperatures (77 K-297 K).


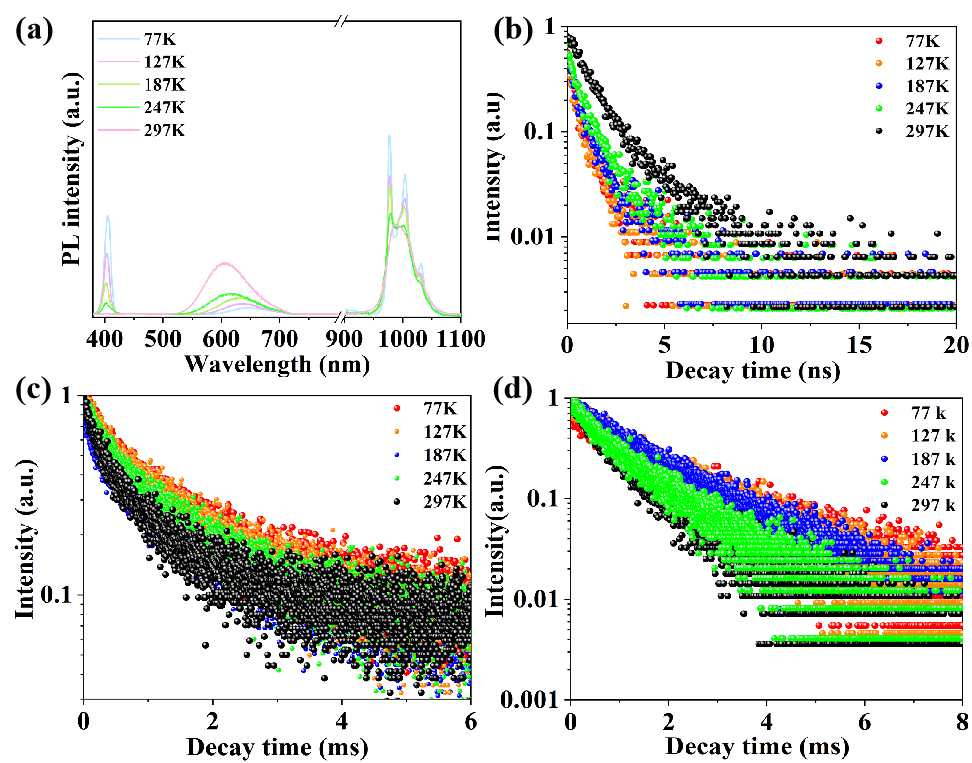


**Figure S17.** (a) Temperature-dependent (77 K-297 K) steady-state PL spectra of CsPbCl_3_:Mn/Yb NCs. Changes in the lifetimes of (b) exciton recombination, (c) Yb emission, and (d) Mn emission in CsPbCl_3_:Mn/Yb NCs under different temperatures (77 K-297 K).


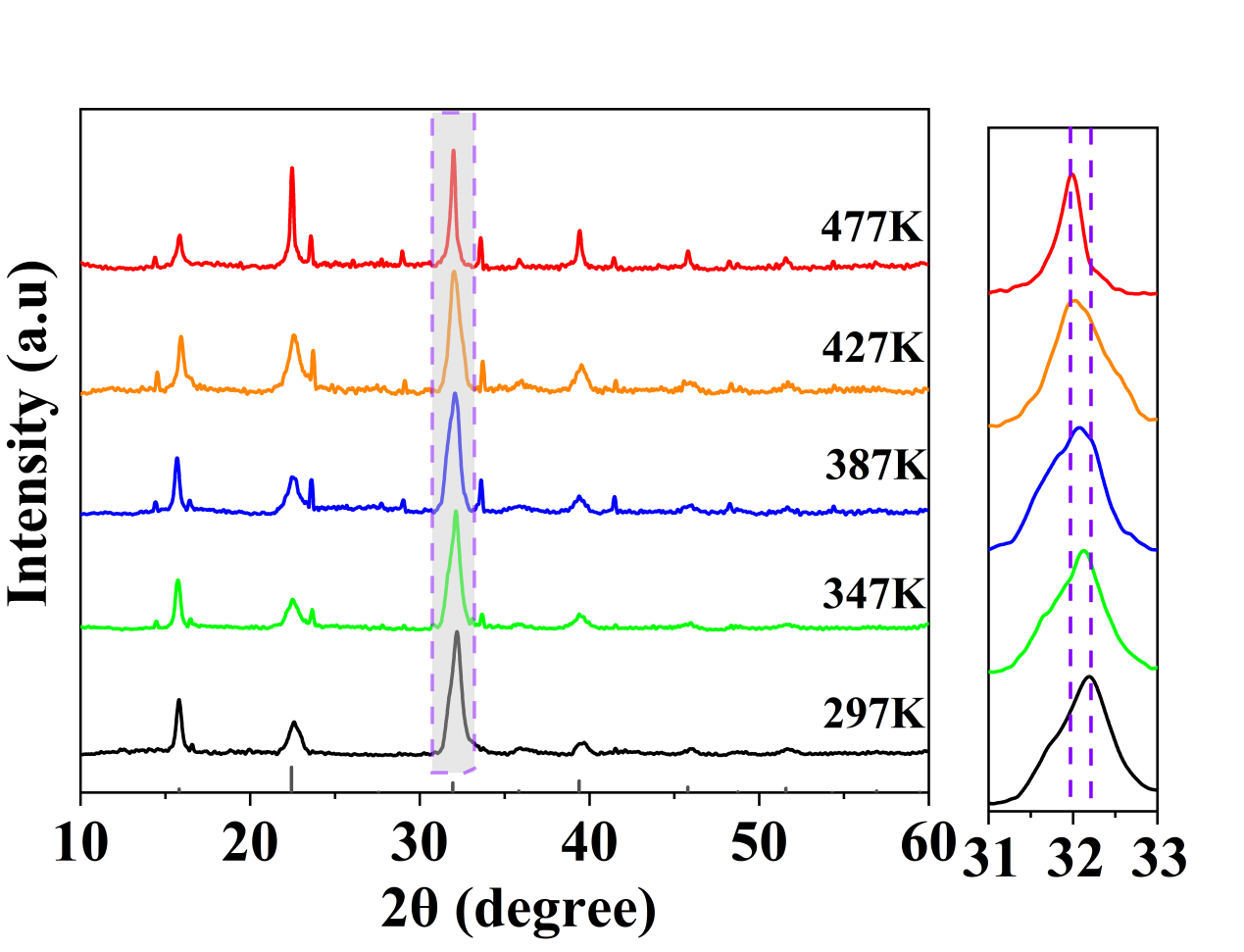


**Figure S18.** XRD patterns of Yb-doped CsPbCl_3_ NCs under different temperatures (297 K-477 K).


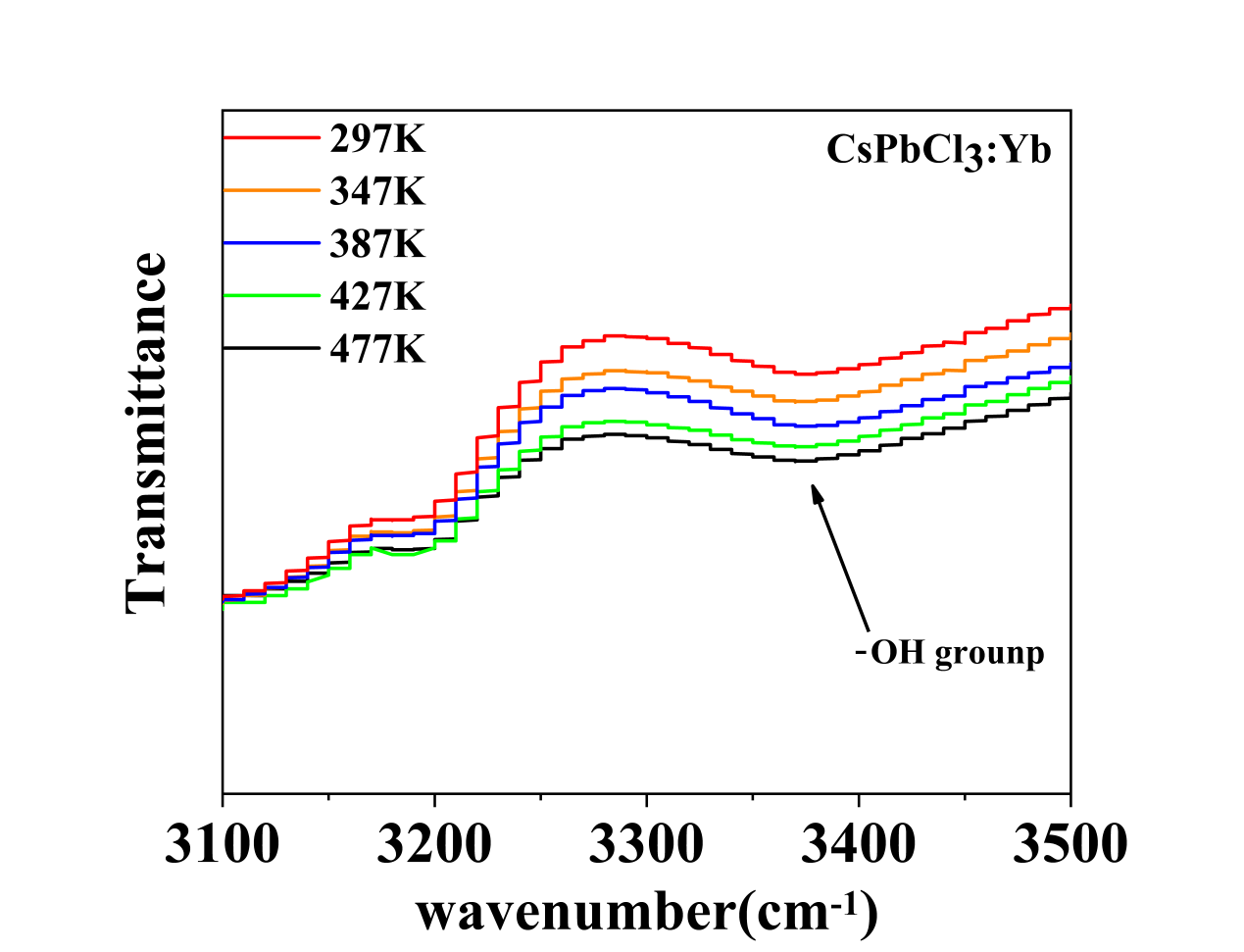


**Figure S19.** Temperature-dependent FTIR spectra of the Yb^3+^-doped CsPbCl_3_ NCs, with the 3150−3400 cm^−1^ peak assigned to −OH absorption (297 K-477 K).


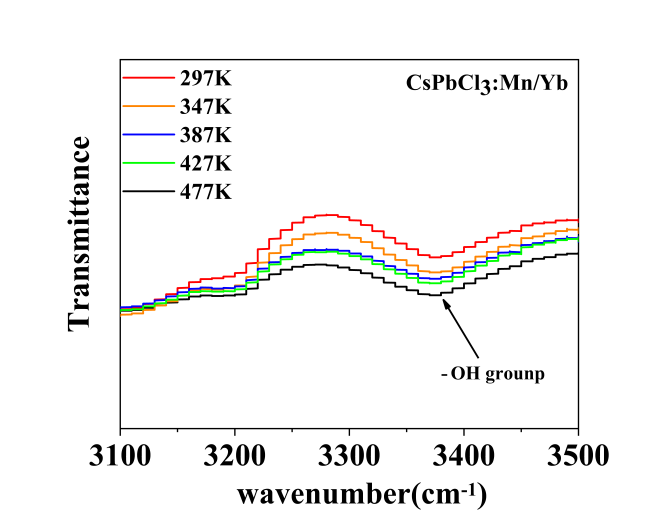


**Figure S20.** Temperature-dependent FTIR spectra of the Yb/Mn-doped CsPbCl_3_ NCs, with the 3150−3500 cm^−1^ peak assigned to −OH absorption (297 K-477 K).


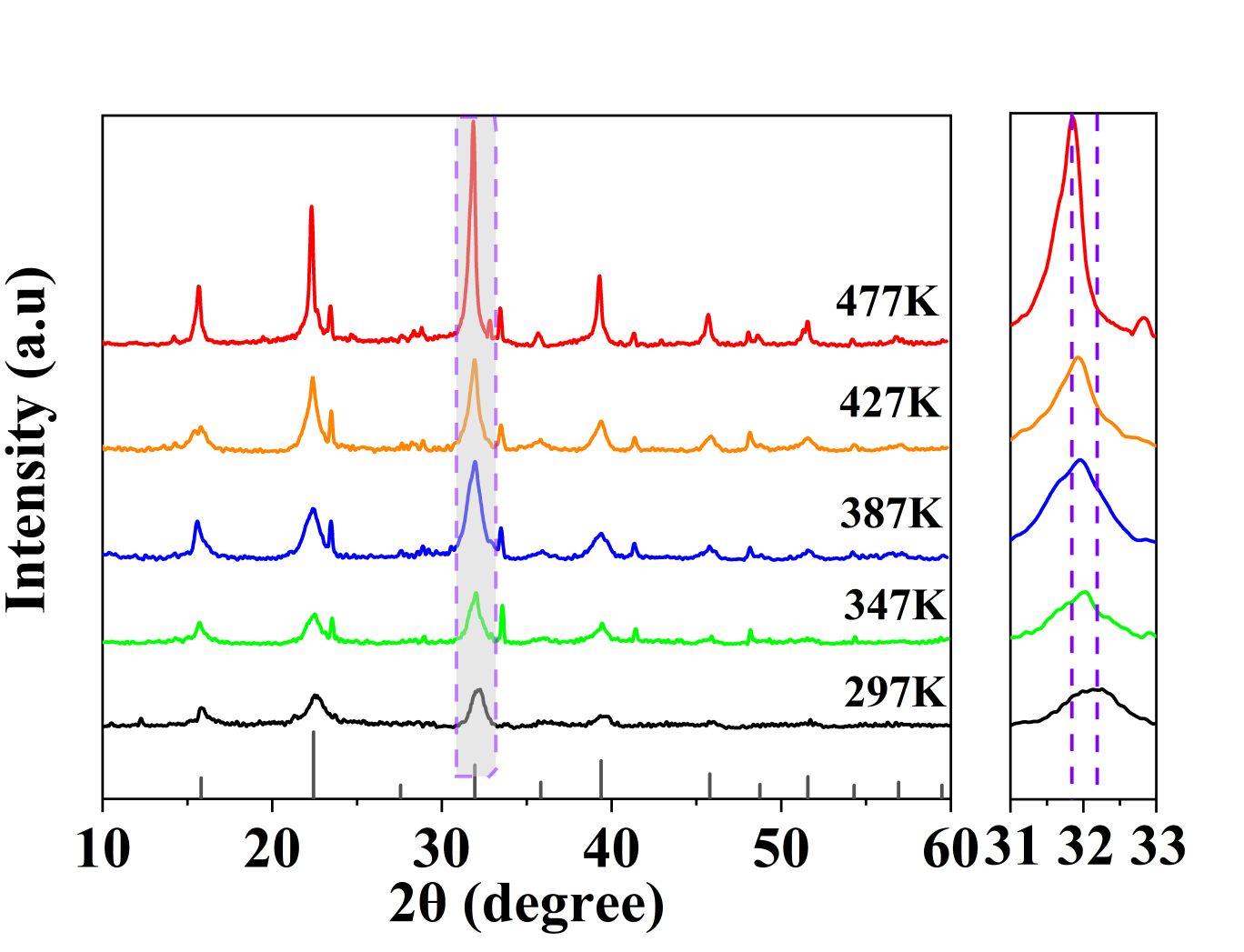


**Figure S21.** XRD patterns of Mn/Yb-doped CsPbCl_3_ NCs under different temperatures (297 K-477 K).


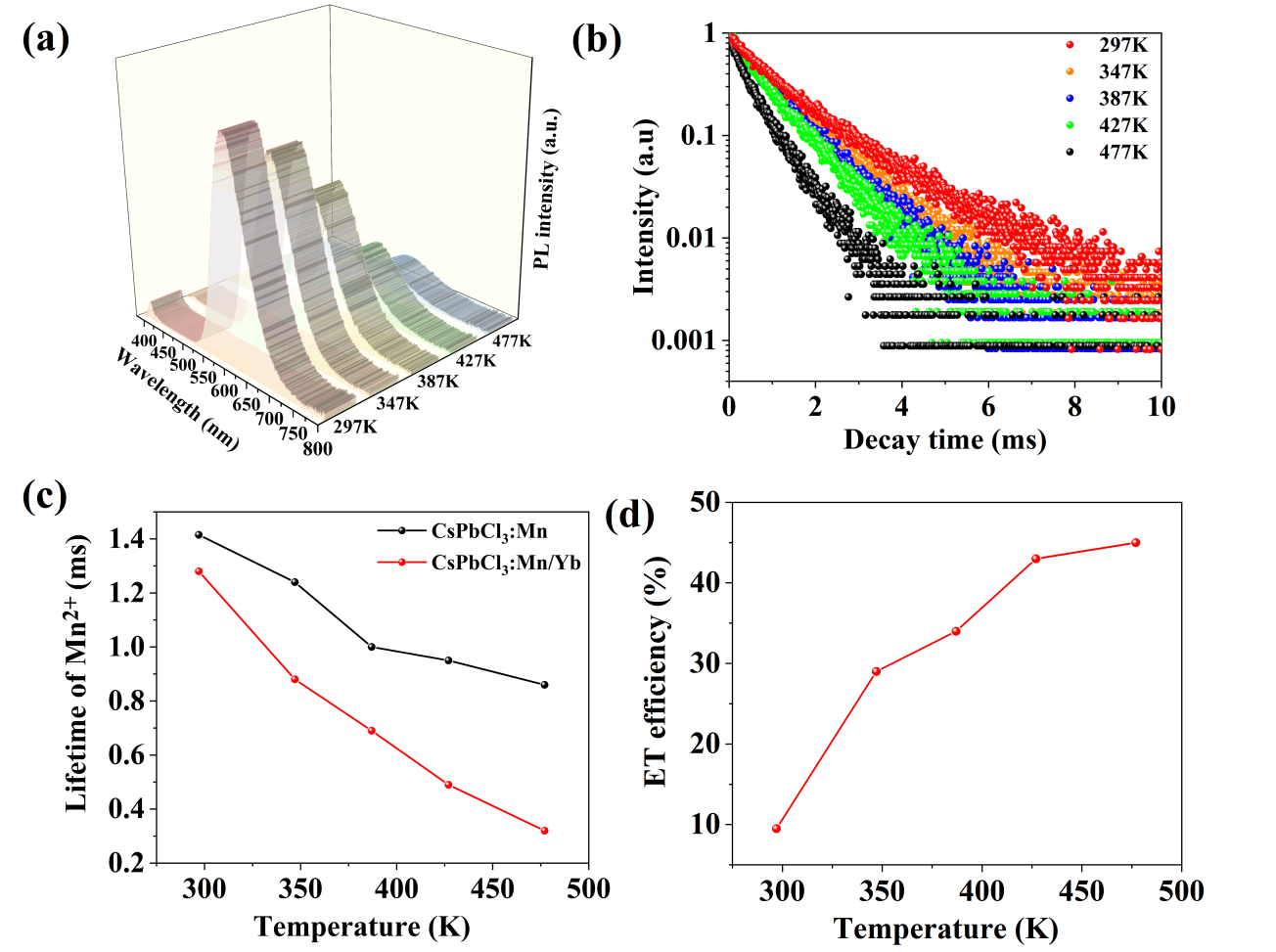


**Figure S22.** (a) Temperature-dependent (297 K−477 K) steady-state PL spectra of CsPbCl_3_:Mn NCs. (b) Lifetime spectra of manganese at different temperatures. (c) Lifetime Variation Plot of Manganese in CsPbCl_3_:Mn and CsPbCl_3_:Mn/Yb at different Temperatures. (d) ET efficiency from Mn to Yb in CsPbCl_3_:Mn/Yb NCs under different temperatures (297 K−477 K).


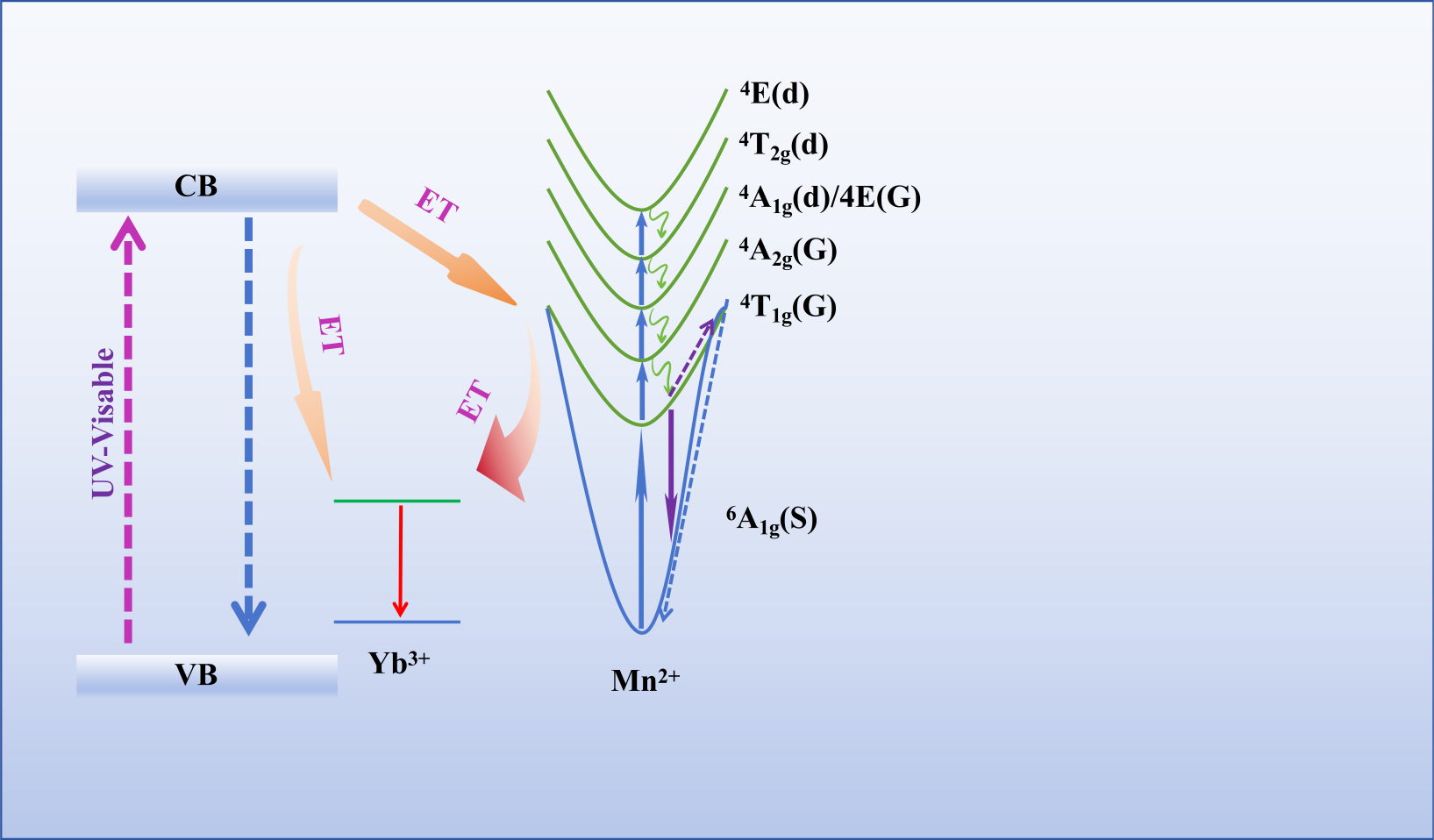


**Figure S23.** Energy level diagram and electron dynamic in CsPbCl_3_: Mn/Yb NCs.

**Table S1** XRD parameters in undoped, Mn-doped, Yb-doped, and Mn/Yb-doped CsPbCl_3_ NCs.

| Samples | Assigned peak/ d (hkl) | Peak position/ ° | d-spacing/ Å | Calculated lattice constant/ Å |
| --- | --- | --- | --- | --- |
| CsPbCl_3_ | (100) | 15.725 | 5.631 | 5.631 |
| CsPbCl_3_:Mn | (100) | 15.951 | 5.550 | 5.550 |
| CsPbCl_3_:Yb | (100) | 15.953 | 5.552 | 5.552 |
| CsPbCl_3_:Mn/Yb | (100) | 15.937 | 5.557 | 5.557 |

**Table S2** The fitted bi-exponential lifetime data for undoped, Mn-doped, Yb-doped, and Mn/Yb-doped CsPbCl_3_NCs.

| Samples | τ_1_ (ns) | A_1_ (%) | τ_2_ (ns) | A_2_ (%) | τ_ave_ (ns) |
| --- | --- | --- | --- | --- | --- |
| CsPbCl_3_ | 0.80 | 80 | 7.51 | 20 | 5.57 |
| CsPbCl_3_:Mn | 0.50 | 72 | 3.43 | 28 | 2.63 |
| CsPbCl_3_:Yb | 0.59 | 92 | 4.43 | 8 | 2.12 |
| CsPbCl_3_:Mn/Yb | 0.49 | 92 | 3.28 | 8 | 1.52 |

**Table S3** The changes in PLQY of excitons, manganese, and ytterbium in CsPbCl_3_, CsPbCl_3_:Mn, CsPbCl_3_:Yb, and CsPbCl_3_:Mn/Yb NCs.

| Sample | PLQY (%) | | |
| --- | --- | --- | --- |
|  | exciton | Mn | Yb |
| CsPbCl_3_ | 6.52 | / | / |
| CsPbCl_3_:Mn | 1.32 | 28.91 | / |
| CsPbCl_3_:Yb | 0.74 | / | 114.43 |
| CsPbCl_3_:Mn/Yb | 0.58 | 16.52 | 100.48 |

**Table S4** XRD parameters of CsPbCl_3_: Mn^2+^/Yb^3+^ NCs prepared with different reaction times.

| Time (min) | Assigned peak/ d (hkl) | Peak position/ ° | d-spacing/ Å | Calculated lattice constant/Å |
| --- | --- | --- | --- | --- |
| 0.5 | (110) | 22.154 | 4.012 | 5.674 |
| 5 | (110) | 22.578 | 3.935 | 5.565 |
| 15 | (110) | 22.581 | 3.932 | 5.561 |
| 30 | (110) | 22.803 | 3.900 | 5.515 |
| 45 | (110) | 22.800 | 3.900 | 5.515 |

**Table S5** The fitted bi-exponential lifetime data for exciton recombination emission in the CsPbCl_3_:Mn/Yb NCs prepared with different reaction times.

| Time (**min**) | τ_1_ (ns) | A_1_ (%) | τ_2_ (ns) | A_2_ (%) | τ_ave_ (ns) |
| --- | --- | --- | --- | --- | --- |
| 0.5 | 0.30 | 79 | 1.64 | 21 | 0.98 |
| 5 | 0.44 | 94 | 2.67 | 6 | 1.13 |
| 15 | 0.48 | 94 | 3.29 | 6 | 1.54 |
| 30 | 0.92 | 72 | 2.48 | 28 | 1.72 |
| 45 | 0.59 | 96 | 3.29 | 4 | 1.51 |

**Table S6** The fitted bi-exponential lifetime data for Mn emission in the CsPbCl_3_:Mn/Yb NCs prepared with different reaction times.

| Time (**min**) | τ_1_ (ms) | A_1_ (%) | τ_2_ (ms) | A_2_ (%) | τ_ave_ (ms) |
| --- | --- | --- | --- | --- | --- |
| 0.5 | 0.14 | 74 | 0.53 | 26 | 0.33 |
| 5 | 0.17 | 37 | 0.52 | 63 | 0.46 |
| 15 | 0.29 | 52 | 0.94 | 48 | 0.78 |
| 30 | 0.29 | 25 | 1.35 | 64 | 1.28 |
| 45 | 0.21 | 35 | 1.06 | 50 | 0.97 |

**Table S7** The fitted bi-exponential lifetime data for Yb emission in the CsPbCl_3_:Mn/Yb NCs prepared with different reaction times.

| Time (**min**) | τ_1_ (ms) | A_1_ (%) | τ_2_ (ms) | A_2_ (%) | τ_ave_ (ms) |
| --- | --- | --- | --- | --- | --- |
| 5 | 0.04 | 50 | 0.51 | 50 | 0.35 |
| 15 | 0.26 | 61 | 0.6 | 33 | 0.45 |
| 30 | 0.39 | 80 | 1.30 | 20 | 0.80 |
| 45 | 0.22 | 46 | 1.17 | 46 | 1.03 |

**Table S8** The fitted bi-exponential lifetime data for exciton recombination emission in the CsPbCl_3_:Mn/Yb NCs prepared with different CCl_4_ contents.

| CCl_4_ content (mL) | τ_1_ (ns) | A_1_ (%) | τ_2_ (ns) | A_2_ (%) | τ_ave_ (ns) |
| --- | --- | --- | --- | --- | --- |
| 1 | 0.73 | 96 | 6.98 | 4 | 2.33 |
| 2 | 0.53 | 90 | 4.2 | 10 | 2.27 |
| 3 | 0.45 | 90 | 3.18 | 10 | 1.66 |
| 4 | 0.48 | 95 | 4.18 | 5 | 1.63 |

**Table S9** The fitted bi-exponential lifetime data for Mn emission in the CsPbCl_3_:Mn/Yb NCs prepared with different CCl_4_ contents.

| CCl_4_ content (mL) | τ_1_ (ms) | A_1_ (%) | τ_2_ (ms) | A_2_ (%) | τ_ave_ (ms) |
| --- | --- | --- | --- | --- | --- |
| 1 | 0.37 | 63 | 1.10 | 37 | 0.79 |
| 2 | 0.3 | 62 | 0.85 | 38 | 0.71 |
| 3 | 0.29 | 65 | 0.47 | 35 | 0.44 |
| 4 | 0.22 | 56 | 0.43 | 44 | 0.35 |

**Table S10** The fitted bi-exponential lifetime data for Yb emission in the CsPbCl_3_:Mn/Yb NCs prepared with different CCl_4_ contents.

| CCl_4_ content (mL) | τ_1_ (ms) | A_1_ (%) | τ_2_ (ms) | A_2_ (%) | τ_ave_ (ms) |
| --- | --- | --- | --- | --- | --- |
| 1 | 0.26 | 60 | 0.79 | 40 | 0.47 |
| 2 | 0.22 | 47 | 1.02 | 53 | 0.89 |
| 3 | 0.52 | 47 | 1.66 | 53 | 1.20 |
| 4 | 0.23 | 6 | 1.54 | 94 | 1.46 |

**Table S11** XRD parameters of CsPbCl_3_: Mn/Yb NCs prepared with different CCl_4_ contents.

| CCl_4_ content (mL) | Assigned peak/ d (hkl) | Peak position/ ° | d-spacing/ Å | Calculated lattice constant/ Å |
| --- | --- | --- | --- | --- |
| 1 | (100) | 16.081 | 5.502 | 5.502 |
| 2 | (100) | 16.177 | 5.481 | 5.481 |
| 3 | (100) | 16.282 | 5.444 | 5.444 |
| 4 | (100) | 16.267 | 5.444 | 5.444 |

**Table S****12** The fitted bi-exponential lifetime data for exciton recombination emission in CsPbCl_3_:Mn/Yb NCs prepared with different Yb-OA contents.

| Yb-OA content (**μL**) | τ_1_ (ns) | A_1_ (%) | τ_2_ (ns) | A_2_ (%) | τ_ave_ (ns) |
| --- | --- | --- | --- | --- | --- |
| 0 | 0.51 | 85 | 3.64 | 15 | 2.28 |
| 10 | 0.62 | 85 | 3.37 | 15 | 1.94 |
| 20 | 0.66 | 79 | 0.66 | 21 | 0.67 |
| 30 | 0.64 | 54 | 0.63 | 46 | 0.65 |
| 40 | 0.45 | 97 | 1.79 | 3 | 0.61 |

**Table S13** The fitted bi-exponential lifetime data for Mn emission in CsPbCl_3_:Mn/Yb NCs prepared with different Yb-OA contents.

| Yb-OA content (**μL**) | τ_1_ (ms) | A_1_ (%) | τ_2_ (ms) | A_2_ (%) | τ_ave_ (ms) |
| --- | --- | --- | --- | --- | --- |
| 0 | 0.30 | 45 | 1.26 | 55 | 1.44 |
| 10 | 0.20 | 35 | 0.80 | 65 | 0.73 |
| 20 | 0.15 | 35 | 0.51 | 65 | 0.47 |
| 30 | 0.64 | 54 | 0.63 | 46 | 0.49 |
| 40 | 0.16 | 52 | 0.43 | 48 | 0.36 |

**Table S14** The fitted bi-exponential lifetime data for Yb emission in CsPbCl_3_:Mn/Yb NCs prepared with different Yb-OA contents.

| Yb-OA content (**μL**) | τ_1_ (ms) | A_1_ (%) | τ_2_ (ms) | A_2_ (%) | τ_ave_ (ms) |
| --- | --- | --- | --- | --- | --- |
| 10 | 0.17 | 38 | 0.57 | 62 | 0.21 |
| 20 | 0.16 | 49 | 0.67 | 51 | 0.24 |
| 30 | 0.09 | 51 | 0.61 | 49 | 0.58 |
| 40 | 0.22 | 54 | 1.17 | 46 | 1.05 |

**Table S15** XRD parameters of CsPbCl_3_:Mn/Yb NCs prepared with different Yb-OA contents.

| Yb-OA content (**μL**) | Assigned peak/ d (hkl) | Peak position/ ° | d-spacing/ Å | Calculated lattice constant/ Å |
| --- | --- | --- | --- | --- |
| 0 | (100) | 15.978 | 5.520 | 5.520 |
| 10 | (100) | 16.162 | 5.480 | 5.480 |
| 20 | (100) | 16.187 | 5.477 | 5.477 |
| 30 | (100) | 16.259 | 5.544 | 5.544 |
| 40 | (100) | 16.306 | 5.432 | 5.432 |

**Table S16** The fitted bi-exponential lifetime data for exciton recombination emission in the CsPbCl_3_:Mn/Yb NCs prepared with different Mn-OA contents.

| Mn-OA content (**μL**) | τ_1_ (ns) | A_1_ (%) | τ_2_ (ns) | A_2_ (%) | τ_ave_ (ns) |
| --- | --- | --- | --- | --- | --- |
| 0 | 0.86 | 88 | 4.99 | 12 | 2.43 |
| 10 | 0.55 | 80 | 3.32 | 20 | 2.18 |
| 20 | 0.6 | 90 | 4.10 | 10 | 2.04 |
| 30 | 0.6 | 99 | 8.27 | 1 | 1.53 |

**Table S17** The fitted bi-exponential lifetime data for Mn emission in the CsPbCl_3_:Mn/Yb NCs prepared with different Mn-OA contents.

| Mn-OA content (**μL**) | | τ_1_ (ms) | A_1_ (%) | | τ_2_ (ms) | | A_2_ (%) | | τ_ave_ (ms) | |  |
| --- | --- | --- | --- | --- | --- | --- | --- | --- | --- | --- | --- |
| 10 | 0.25 | | | 35 | | 0.68 | | 65 | | 0.63 | |
| 20 | 0.21 | | | 44 | | 0.57 | | 56 | | 0.49 | |
| 30 | 0.18 | | | 51 | | 0.49 | | 49 | | 0.39 | |

**Table S18** The fitted bi-exponential lifetime data for Yb emission in the CsPbCl_3_:Mn/Yb NCs prepared with different Mn-OA contents.

| Mn-OA content (**μL**) | τ_1_ (ms) | A_1_ (%) | τ_2_ (ms) | A_2_ (%) | τ_ave_ (ms) |
| --- | --- | --- | --- | --- | --- |
| 0 | 0.21 | 41 | 1.09 | 59 | 1.78 |
| 10 | 0.21 | 58 | 0.99 | 42 | 0.83 |

**Table S19** XRD parameters of CsPbCl_3_:Mn/Yb NCs prepared with different Mn-OA contents.

| Mn-OA content (**μL**) | Assigned peak/ d (hkl) | Peak position/ ° | d-spacing/ Å | Calculated lattice constant/ Å |
| --- | --- | --- | --- | --- |
| 0 | (100) | 15.973 | 5.544 | 5.544 |
| 10 | (100) | 16.232 | 5.463 | 5.463 |
| 20 | (100) | 16.340 | 5.425 | 5.425 |
| 30 | (100) | 16.310 | 5.425 | 5.425 |

**Table S20** The fitted bi-exponential lifetime data for exciton recombination emission in the CsPbCl_3_:Yb NCs under different temperatures (77 K-297 K).

| Temperature (K) | τ_1_ (ns) | A_1_ (%) | τ_2_ (ns) | A_2_ (%) | τ_ave_ (ns) |
| --- | --- | --- | --- | --- | --- |
| 77 | 2.28 | 5 | 0.30 | 95 | 0.86 |
| 127 | 0.50 | 95 | 2.67 | 5 | 0.98 |
| 187 | 0.56 | 89 | 3.68 | 11 | 1.97 |
| 247 | 0.71 | 75 | 2.97 | 25 | 2.02 |
| 297 | 0.56 | 83 | 4.56 | 17 | 3.06 |

**Table S21**The fitted bi-exponential lifetime data for Yb emission in the CsPbCl_3_:Yb NCs under different temperatures (77 K-297 K).

| Temperature (K) | τ_1_ (ms) | A_1_ (%) | τ_2_ (ms) | A_2_ (%) | τ_ave_ (ms) |
| --- | --- | --- | --- | --- | --- |
| 77 | 0.15 | 53 | 1.74 | 47 | 1.59 |
| 127 | 0.26 | 46 | 1.70 | 54 | 1.54 |
| 187 | 0.23 | 42 | 1.64 | 58 | 1.50 |
| 247 | 0.27 | 49 | 1.44 | 51 | 1.25 |
| 297 | 0.22 | 50 | 1.21 | 50 | 1.06 |

**Table S22** The fitted bi-exponential lifetime data for exciton recombination emission in the CsPbCl_3_:Mn/Yb NCs under different temperatures (77 K-297 K).

| Temperature (K) | τ_1_ (ns) | A_1_ (%) | τ_2_ (ns) | A_2_ (%) | τ_ave_ (ns) |
| --- | --- | --- | --- | --- | --- |
| 77 | 1.63 | 12 | 0.30 | 88 | 0.87 |
| 127 | 0.28 | 56 | 1.42 | 44 | 1.19 |
| 187 | 0.37 | 57 | 1.48 | 43 | 1.21 |
| 247 | 0.35 | 50 | 1.57 | 50 | 1.34 |
| 297 | 1.12 | 86 | 4.66 | 14 | 2.47 |

**Table S23** The fitted bi-exponential lifetime data for Mn emission in the CsPbCl_3_:Mn/Yb NCs under different temperatures (77 K-297 K).

| Temperature (K) | τ_1_ (ms) | A_1_ (%) | τ_2_ (ms) | A_2_ (%) | τ_ave_ (ms) |
| --- | --- | --- | --- | --- | --- |
| 77 | 0.05 | 33 | 1.54 | 67 | 1.53 |
| 127 | 0.22 | 29 | 1.53 | 71 | 1.51 |
| 187 | 0.43 | 31 | 1.52 | 69 | 1.40 |
| 247 | 0.09 | 34 | 1.45 | 66 | 1.38 |
| 297 | 0.30 | 26 | 1.33 | 74 | 1.25 |

**Table S24** The fitted bi-exponential lifetime data for Yb emission in the CsPbCl_3_:Mn/Yb NCs under different temperatures (77 K-297 K).

| Temperature (K) | τ_1_ (ms) | A_1_ (%) | τ_2_ (ms) | A_2_ (%) | τ_ave_ (ms) |
| --- | --- | --- | --- | --- | --- |
| 77 | 0.37 | 53 | 1.69 | 47 | 1.42 |
| 127 | 0.35 | 49 | 1.63 | 51 | 1.40 |
| 187 | 0.22 | 64 | 1.57 | 36 | 1.29 |
| 247 | 0.32 | 53 | 1.39 | 47 | 1.17 |
| 297 | 0.22 | 59 | 1.10 | 41 | 0.90 |

**Table S25** The fitted bi-exponential lifetime data for Yb emission in the CsPbCl_3_:Yb NCs under different temperatures (297 K-477 K).

| Temperature (K) | τ_1_ (ms) | A_1_ (%) | τ_2_ (ms) | A_2_ (%) | τ_ave_ (ms) |
| --- | --- | --- | --- | --- | --- |
| 297 | 0.22 | 50 | 1.21 | 50 | 1.06 |
| 347 | 0.22 | 54 | 1.47 | 46 | 1.33 |
| 387 | 0.33 | 52 | 1.55 | 48 | 1.40 |
| 427 | 0.22 | 62 | 1.29 | 38 | 1.05 |
| 477 | 0.34 | 60 | 1.34 | 40 | 1.07 |

**Table S26** The fitted bi-exponential lifetime data for exciton recombination emission in the CsPbCl_3_:Yb NCs under different temperatures (297 K-477 K).

| Temperature (K) | τ_1_ (ns) | A_1_ (%) | τ_2_ (ns) | A_2_ (%) | τ_ave_ (ns) |
| --- | --- | --- | --- | --- | --- |
| 297 | 0.56 | 83 | 4.56 | 17 | 3.06 |
| 347 | 0.63 | 85 | 4.03 | 15 | 2.43 |
| 387 | 0.56 | 89 | 3.68 | 11 | 2.00 |
| 427 | 0.32 | 86 | 1.36 | 14 | 0.76 |
| 477 | 0.46 | 88 | 1.31 | 12 | 0.69 |

**Table S27** The fitted bi-exponential lifetime data for exciton recombination emission in the CsPbCl_3_:Mn/Yb NCs under different temperatures (297 K-477 K).

| Temperature (K) | τ_1_ (ns) | A_1_ (%) | τ_2_ (ns) | A_2_ (%) | τ_ave_ (ns) |
| --- | --- | --- | --- | --- | --- |
| 297 | 1.12 | 86 | 4.33 | 14 | 2.36 |
| 347 | 1.25 | 95 | 5.45 | 5 | 2.03 |
| 387 | 0.47 | 69 | 1.86 | 31 | 1.36 |
| 427 | 0.45 | 88 | 2.19 | 12 | 1.14 |
| 477 | 0.23 | 80 | 1.27 | 20 | 0.75 |

**Table S28** The fitted bi-exponential lifetime data for Yb emission in the CsPbCl_3_:Mn/Yb NCs under different temperatures (297 K-477 K).

| Temperature (K) | τ_1_ (ms) | A_1_ (%) | τ_2_ (ms) | A_2_ (%) | τ_ave_ (ms) |
| --- | --- | --- | --- | --- | --- |
| 297 | 0.22 | 60 | 1.10 | 40 | 0.89 |
| 347 | 0.27 | 48 | 1.16 | 52 | 1.03 |
| 387 | 0.28 | 48 | 1.17 | 52 | 1.07 |
| 427 | 0.36 | 48 | 1.24 | 52 | 1.09 |
| 477 | 0.23 | 57 | 1.05 | 43 | 0.86 |

**Table S29** The fitted bi-exponential lifetime data for Mn emission in the CsPbCl_3_:Mn/Yb NCs under different temperatures (297 K-477 K).

| Temperature (K) | τ_1_ (ms) | A_1_ (%) | τ_2_ (ms) | A_2_ (%) | τ_ave_ (ms) |
| --- | --- | --- | --- | --- | --- |
| 297 | 0.30 | 28 | 1.33 | 72 | 1.25 |
| 347 | 0.28 | 54 | 1.04 | 46 | 0.88 |
| 387 | 0.12 | 59 | 0.80 | 41 | 0.69 |
| 427 | 0.08 | 63 | 0.58 | 37 | 0.49 |
| 477 | 0.15 | 54 | 0.39 | 46 | 0.32 |

**Table S30** Temperature stability of near-infrared luminescence of different materials.

| Phosphors | Temperature (K) | λ_em_ (nm) | Norm. PL intensity | Ref. |
| --- | --- | --- | --- | --- |
| **Ca_2_LuZr_2_Al_3_O1_2_:Cr^3+^,Yb^3+^** | 427 | 780 | 0.69 fold | [5] |
| LuCa_2_ScZrGa_2_GeO1_2_:Cr^3+^ | 423 | 750 | 0.70 fold | [6] |
| CsPbCl_3_:Er^3+^,Yb^3+^ | 356 | 980 | 1.10 fold | [7] |
| Mg_4_Ta_2_O_9_:Ni ^2+^ | 473 | 1300 | 0.73 fold | [8] |
| YGa_3_(BO_3_)_4_: Cr^3+^ | 427 | 770 | 0.8 fold | [9] |
| CsPbCl_3_:Mn/Yb | 427 | 980 | 3.2 fold | This work |

References

1. Kresse, G. G.; J.J. Furthmüller, Efficient Iterative Schemes for Ab Initio Total-Energy Calculations Using a Plane-Wave Basis Set, Phys. Rev. B, 1996, 54, 11169.

2. Blöchl, P.E., Projector augmented-wave method, Phys. Rev. B, 1994, 50, 17953-17979.

3. Perdew, J.P.; Burke, K.; Ernzerhof, M., Generalized Gradient Approximation Made Simple, Phys. Rev. L, 1996, 77, 3865-3868.

4. Hammer, B.; Hansen, L.B.; Nørskov, J.K.; Improved adsorption energetics within density-functional theory using revised Perdew-Burke-Ernzerhof functionals, Phys. Rev. B, 1999, 59, 7413-7421.

5. Yang, Q.; Wu, X.; Xu, Y.; Zhang, X. C.; Zhong, S.; Yin, C.; Wang, L.; Zhou, H. You, Efficient broadband NIR garnet phosphor Ca_2_LuScGa_2_Si_2_O_12_:Cr^3+^,Yb^3+^ for NIR pc-LED applications, Materials Today Chemistry, 2024, 37.

6 . Xie, J.; Tian, J.; Zhuang, W.; Near-Infrared LuCa_(2)_ScZrGa_(2)_GeO_(12)_:Cr^(3+)^ Garnet Phosphor with Ultra-broadband Emission for NIR LED Applications, Inorg. Chem., 2023, 62, 10772-10779.

7. Li, D.; Chen, G.; Near-Infrared Photoluminescence from Ytterbium- and Erbium-Codoped CsPbCl_3_ Perovskite Quantum Dots with Negative Thermal Quenching, The Journal of Physical Chemistry Letters, 2023, 14, 2837-2844.

8. Li, J.; Wang, C.; Niu, Y.; Wang, Y.; Wu, F.; Qi, Z.; Teng, Y.; Dong, H.; Mu, Z.; Efficient broad-band NIR-II emitting phosphor Mg_4_Ta_2_O_9_: Ni^2+^ with satisfactory thermal stability of luminescence, Ceramics International, 2024, 50, 18647-18654.

9. Zou, Y.; Hu, C.; Lv, S.; Shao, Y.; Teng, B.; You, F.; Xu, H.; Zhong, D.; Realization of Broadband Near-Infrared Emission with High Thermal Stability in YGa_(3)_(BO_(3)_)(4): Cr_(3+)_ Borate Phosphor, Inorg. Chem., 2023, 62, 19507-19515.
